# The PM20D1-OLE pathway induces microglia rewiring to ameliorate Alzheimer disease

**DOI:** 10.1038/s41419-026-08791-1

**Published:** 2026-04-27

**Authors:** Victoria Pozzi-Ruiz, Aida Giner de Gracia, Liliane Glauser, Mario Romani, Fatima Gunter-Rahman, Alejandro González-Ramón, Mahmood Haj-Yahya, Rajasekhar Kolla, Allison M. Burns, Hilal A. Lashuel, Johan Auwerx, Johannes Gräff, Jose V. Sanchez-Mut

**Affiliations:** 1 Laboratory of Functional Epi-Genomics of Aging and Alzheimer’s disease, Instituto de Neurociencias, Universidad Miguel Hernández-Consejo Superior de Investigaciones Científicas (UMH-CSIC), Alicante, Spain; 2 Laboratory of Neuroepigenetics, School of Life Sciences, Brain Mind Institute, École Polytechnique Fédérale de Lausanne, Lausanne, Switzerland; 3 Laboratory of Integrative Systems Physiology, Institute of Bioengineering, École Polytechnique Fédérale de Lausanne, Lausanne, Switzerland; 4 Laboratory of Molecular and Chemical Biology of Neurodegeneration, School of Life Sciences, Brain Mind Institute, École Polytechnique Fédérale de Lausanne, Lausanne, Switzerland

**Keywords:** Alzheimer's disease, Transcriptomics

## Abstract

There is increasing evidence of microglia participation in Alzheimer’s disease (AD), which incentives their modulation to intercept the disease. Here, we describe a new mechanism by which the recently AD-associated Peptidase M20 Domain Containing 1 (PM20D1) instructs microglia to tackle AD. We show that the PM20D1-derived N-oleoyl-Leucine (OLE) improves AD pathologies in two animal models of AD. OLE induces microglia association with amyloid beta (Aβ) plaques, reduce their size, number and toxicity, and leads to enhanced neuroprotection and cognition. Furthermore, OLE also increases Aβ chemotaxis and clearance in microglia cultures and enhances cell viability in neurons subjected to AD-related stressors. Finally, we also find evidence for a PM20D1- and OLE-mediated microglia association with amyloid plaques and neuroprotection in human AD brains. In sum, our results provide further insight into the protective role of PM20D1 in AD and support the use of OLE as a microglia-modifying treatment for AD.

## Introduction

Multiple lines of evidence suggest a prominent role of microglia in Alzheimer’s disease (AD). Genetic risk variants are enriched in microglia-regulatory regions [1, 2] and microglia transcriptional programs prominently altered in the disease [3]. As AD progresses, microglia proliferate and associates with amyloid plaques [4, 5]. They lose homeostatic and amyloid beta (Aβ) clearance capacities and acquire pro-inflammatory and neurotoxic traits [6]. Both protective and detrimental effects of microglia have been described in AD [7], but little is known about the mechanisms that regulate their behavior at different stages of the disease.

Recently, we and others have identified a new AD risk region centered on the Peptidase M20 Domain Containing 1 (*PM20D1*) [8–13]. *PM20D1* is a quantitative trait locus (QTL) that has also been associated with diabetes [14, 15] and obesity [14, 16–19] among other diseases [20–31]. Human low expression carriers of *PM20D1* have a higher risk to develop metabolic disorders [14, 16–19] and AD [8–13], while *PM20D1* overexpression in mouse models improves glucose metabolism [14, 16] and reduces amyloid levels and cognitive deficits [8]. PM20D1 is a secreted enzyme that regulates the conjugation of fatty acids with amino acids, generating a series of compounds named N-acyl amino acids (NAAA) [14]– also known as elmiric and lipo-amino acids. NAAA are present in multiple tissues, including brain [32, 33], and their levels are directly correlated with the expression of PM20D1 in humans [34] and mice [14]. They are believed to act as protective molecules regulating inflammation, ischemia and traumatic brain injury [35–40]. However, the precise mechanisms by which NAAA exert such effects are not well understood, let alone their potential role in AD.

Seeking to address this question, we investigated the effects of *PM20D1*-derived NAAA on the onset and progression of AD, by combining a set of behavioral, histochemical and molecular techniques in animal models and human samples of AD. We found that the exogenous administration of NAAA in a *C. elegans* model of AD alleviates motility deficits and amyloid aggregation and that its supplementation in an AD mouse model ameliorates cognitive deficits and amyloid levels. In line with this, NAAA treatment increases microglia association with amyloid plaques, reducing their number, size and toxicity, and improves neuronal fitness. Mechanistically, NAAA induce cell-type-specific transcriptional programs that result in increased expression of amyloid-binding genes in microglia and genes associated with the unfolded-protein response in neuronal cells. These changes are functionally associated with an increase in Aβ chemotaxis and clearance in primary microglia, and enhanced neuronal cell viability under reactive oxygen species (ROS) and unfolded-protein stress. In support of this, we found that NAAA-induced genes in human samples are positively and negatively correlated with specific transcriptional signatures associated with cognitive reserve and neurofibrillary tangles (NFT), respectively. Taken together, these results provide unprecedented molecular detail on the functional relevance of a recently discovered AD risk factor, and supports the use of NAAA as potential disease-modifying agents for the treatment of AD.

## Results

### NAAA improve AD phenotypes and pathologies in a C. elegans and mouse model of AD

To explore the potential role of NAAA in AD, we focused on OLE (N-Oleoyl-Leucine, Supplementary Fig. 1), an established metabolic product of PM20D1 whose supplementation has been shown to be well tolerated in vivo [14, 15]. We took advantage of two different AD models. The GMC101 strain of *C. elegans*, which aggregates Aβ peptides in the body wall of muscle cells after a temperature shift from 20 to 25 °C, impairing their mobility and viability [41], and the APP/PS1 mouse model, which shows a progressive accumulation of amyloid plaques, astrogliosis as well as learning and memory deficits [42, 43].

**Fig. 1:**
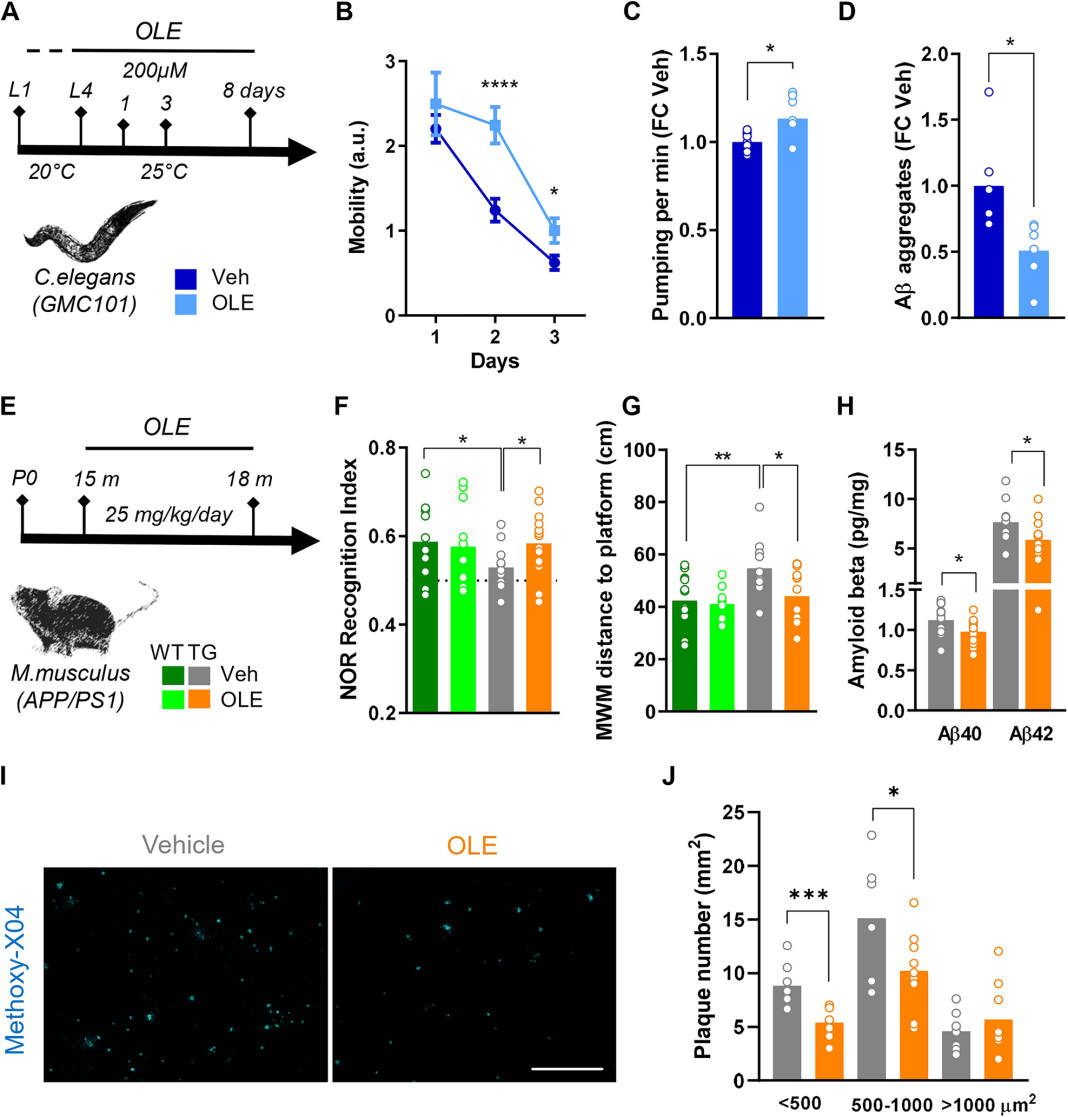
AD pathologies and phenotypes in OLE treated GMC101 worms and APP/PS1 mice. **A** Schematics of OLE treatment in GMC101 worms. OLE was provided from day 1 to day 8 of adulthood. L1 and L4, larval stages 1 and 4, respectively. **B** Mobility assessment in vehicle and OLE treated GMC101 worms during the 3 first days of adulthood. a.u., arbitrary units. n = 73-100 worms per time, *p* < 0.0001 and *p* = 0.0152 for day 2 and 3, respectively; one-sided Student’s t-test. **C** Pumping per minute in vehicle and OLE treated GMC101 worms at day 3. *n* = 7 per group, *p* = 0.0162; one-sided *t*-test. **D** Relative amyloid aggregation in vehicle and OLE treated GMC101 worms at day 1. *n* = 6 experiments of ~1 000 worms each, pval=0.0108; one-sided Student’s *t*-test. **E** Schematics of OLE treatment in APP/PS1 mice. OLE was provided from 15 to 18 months (m) of age. P0, postnatal day 0. **F** Novel object recognition (NOR) performance of vehicle and OLE treated APP/PS1 mice. *n* = 11–15 per group, *p* = 0.0250 for TG vehicle vs WT vehicle and *p* = 0.0147 for TG treatment vs TG vehicle; one-sided Student’s t-test. **G** Morris Water Maze (MWM) memory performance of vehicle and OLE treated APP/PS1 mice. *n* = 9–13 per group, *p* = 0.0067 for TG vehicle vs WT vehicle and *p* = 0.0150 for TG treatment vs TG vehicle; one-sided Student’s *t*-test. **H** ELISA of frontal cortex samples from vehicle and OLE treated APP/PS1 mice. *n* = 12 and 15, respectively, and *p* = 0.0188 for Aβ40 and *p* = 0.0134 for Aβ42; one-sided Student’s *t*-test. **I** Representative cortex methoxy-X04 (MX04) IHC amyloid plaque staining images of vehicle and OLE treated APP/PS1 mice. Scale bar, 300 µm. **J** Amyloid plaque number quantification of (**I**). *n* = 7–9 per group, *p* = 0.0009 and 0.0225, for small and medium size plaques, respectively; one-sided Student’s *t*-test. Single values (animals) are represented by dots. FC, fold change. *p < 0.05, **p < 0.01, and ***p < 0.001.

We synthesized OLE (Supplementary Fig. 1) and investigated the most effective OLE dose in *C.elegans* by measuring dosage effects on viability. We observed that 200 μM significantly reduced the percentage of worms’ death after one week of treatment (Supplementary Fig. 2a). Accordingly, we found that this dose also improved both, AD-like phenotypes – i.e., spontaneous and induced mobility [44] (Fig. 1A–C) – and amyloid pathologies (Fig. 1D, and Supplementary Fig. 3).

We next sought to determine the most effective OLE dose in APP/PS1 mice. We measured the dosage effect on body weight since daily intraperitoneal (i.p.) administration of OLE has been shown to reduce body weight [14, 15]. We observed that OLE 50 mg/kg/day, but not 25 mg/kg/day per oral (p.o.) administration induced a significant loss of body weight after two weeks of treatment (Supplementary Fig. 2b). We reasoned that doses inducing a significant loss of body weight would not be suitable for sustained treatments and hence selected the lower dose for subsequent experiments (Fig. 1E). As expected [45–47], OLE was absorbed and crossed the blood brain barrier (Supplementary Fig. 2c,d). We treated symptomatic APP/PS1 mice with strong cognitive deficits and pathology levels (i.e., fifteen-months old) [43] for three months and subsequently evaluated their memory performance and AD pathologies. No differences in water consumption and/or body weight were observed after three months of treatment (Supplementary Fig. 2e,f). However, we found that APP/PS1 mice receiving OLE showed improved memory performance as measured by the novel object recognition (NOR) (Fig. 1F) and the Morris Water Maze (MWM) tests (Fig. 1G and Supplementary Fig. 4a), with no significant differences in the MWM learning phase (Supplementary Fig. 4b). No significant differences were observed in wild-type (WT) treated mice (Fig. 1F, G, and Supplementary Fig. 4a, b). Remarkably, APP/PS1 memory improvement was also accompanied by a significant reduction in the number of plaques (Fig. 1I, J), mainly in small- and medium-size amyloid plaques as measured by immunohistochemistry (IHC) staining. This decrease was paralleled by a reduction in the levels of Aβ 40 and Aβ 42 (Fig. 1H), measured by ELISA, and was not related to altered APP expression or processing (Supplementary Fig. 5a-c). Taken together, these results indicate that OLE treatment increases Aβ clearance and improves AD phenotypes in both *C. elegans* and mouse models of AD, with no significant effects in WT littermates.

### OLE treatment in APP/PS1 mice induces cell type-specific gene expression changes, predominantly in microglia

**Fig. 2:**
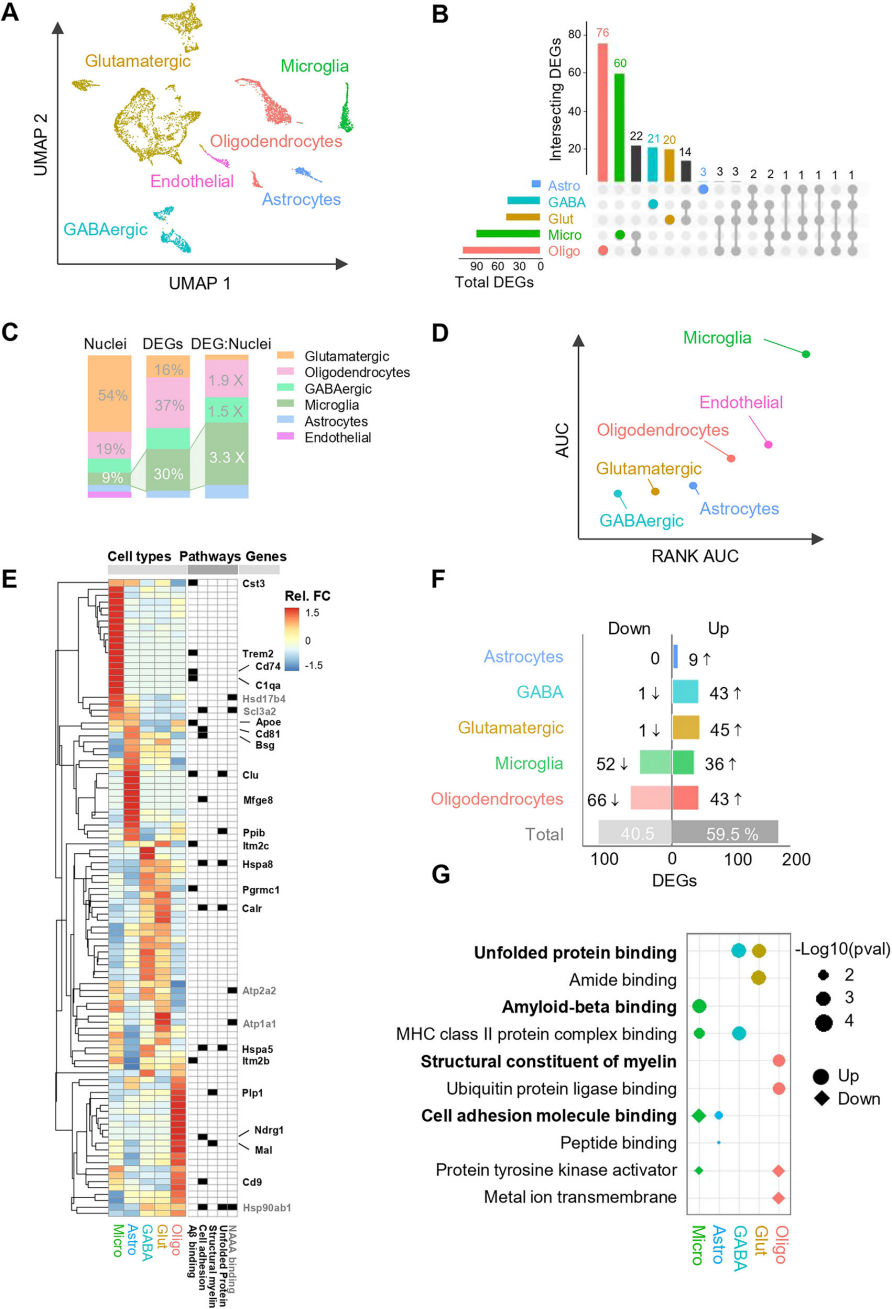
Single-nuclei RNA sequencing of OLE treated APP/PS1 mice. **A** UMAP clustering of entorhinal cortex samples obtained from vehicle and OLE treated APP/PS1 mice colored by cell type. **B** UpSet plot showing differentially expressed genes (DEGs) per cell type. **C** Proportion of cell-type nuclei, DEG and the ratio of both in our dataset. **D** Area under the curve (AUC) resulting from the Augur Cell type prioritization method. **E** Heatmap clustering of relative fold change (Rel. FC) gene (rows) between cell types (columns) of top 20 DEGs per cell type after OLE treatment. Genes representative of enriched pathways and/or coding for NAAA-interacting proteins are indicated. Bold text indicates pathways in (**G**). **F** Distribution of up- and down-regulated DEGs per cell type and overall. **G** Two most significative up- and down-regulated gene ontology (GO) terms per cell type.

To better understand the mechanisms by which OLE protects against AD, we next applied 10X single-nuclei RNA sequencing (snRNAseq) to characterize its effects on specific cellular compartments in the cortex of APP/PS1 mice. A total of 12,918 nuclei (6,745 and 6,173 in vehicle and OLE treated APP/PS1 mice, respectively) from entorhinal cortex (ECX, periallocortex), one of the most susceptible regions to AD in humans [48] and APP/PS1 mice [49], were sequenced with Illumina HiSeq 4000 (average 3178 genes per nuclei). We identified fifteen clusters corresponding to six different cell types (Fig. 2A and Supplementary Fig. 6a) and found 231 differentially expressed genes (DEGs) and enriched pathways between treated and non-treated cells (Supplementary Tables 1–7). A significant fraction of the DEGs was cell-type specific (82%), and mostly found in oligodendrocytes (34.5%) followed by microglia (27.3%), GABAergic (9.5%) and Glutamatergic (9.1%) cells (Fig. 2B).

We then determined the most responsive cell types using two alternative methods. First, we considered the number of DEGs in relation to the number of cells per cell type, since both are highly correlated, even with modest transcriptional changes [50]; second, we applied the Augur [50] cell type prioritization method, which uses a random forest classifier to rank cell types irrespective of their cluster size and/or feature distribution. Both the relative DEG enrichment and the Augur method identified microglia as the most responsive cell type to OLE treatment (Fig. 2c, d). Gene Ontology (GO) up-regulated terms were consistent with the observed OLE induced effects on pathology: amyloid-clearance in microglia (“Amyloid-beta binding” GO:0001540, adj.pval 4.0 × 10^−5^), myelinization in oligodendrocytes (“Structural constituent of myelin sheath” GO:0019911, adj.pval 2.7 × 10^−4^) and neuroprotection in neuronal cells (“Unfolded protein binding” GO:0051082, adj.pval 2.5 × 10^−5^ and 4.2 × 10^−5^ in GABAergic and Glutamatergic cells, respectively) (Fig. 2E–G and Supplementary Table 7). AD-related genes such as Apolipoprotein E (*Apoe*), the main genetic risk factor of AD [48], Cystatin C (*Cst3*) and Integral Membrane Protein 2B (*Itm2b*), whose overexpression protect against AD [51, 52], and Hydroxysteroid 17-β Dehydrogenase 4 (*Hsd17b4*) [53] and Solute Carrier Family 3 Member 2 (*Slc3a2*) [54], which regulate amyloid clearance and phagocytosis, were particularly up-regulated in microglia. Other pathology-relevant genes such as Calreticulin (*Calr*), which reduces amyloid toxicity [55], ATPase sarcoplasmic/endoplasmic reticulum Ca2+ transporting 2 (*Atp2a2*), which regulates autophagy [56], and Heat Shock Protein 90 alpha family class B member 1 (*Hsp90ab1*) and Family A Member 5 (*Hspa5*), which protect against unfolded-protein stress and learning and memory deficits [57, 58], were mostly up-regulated in GABAergic and glutamatergic cells (Fig. 2E and Supplementary Tables 1–6). Collectively, these findings suggest that OLE treatment induces cell-type-specific RNA expression changes, mostly affecting microglia, with a putative neuroprotective effect.

### OLE treatment increases microglia association with amyloid plaques

**Fig. 3:**
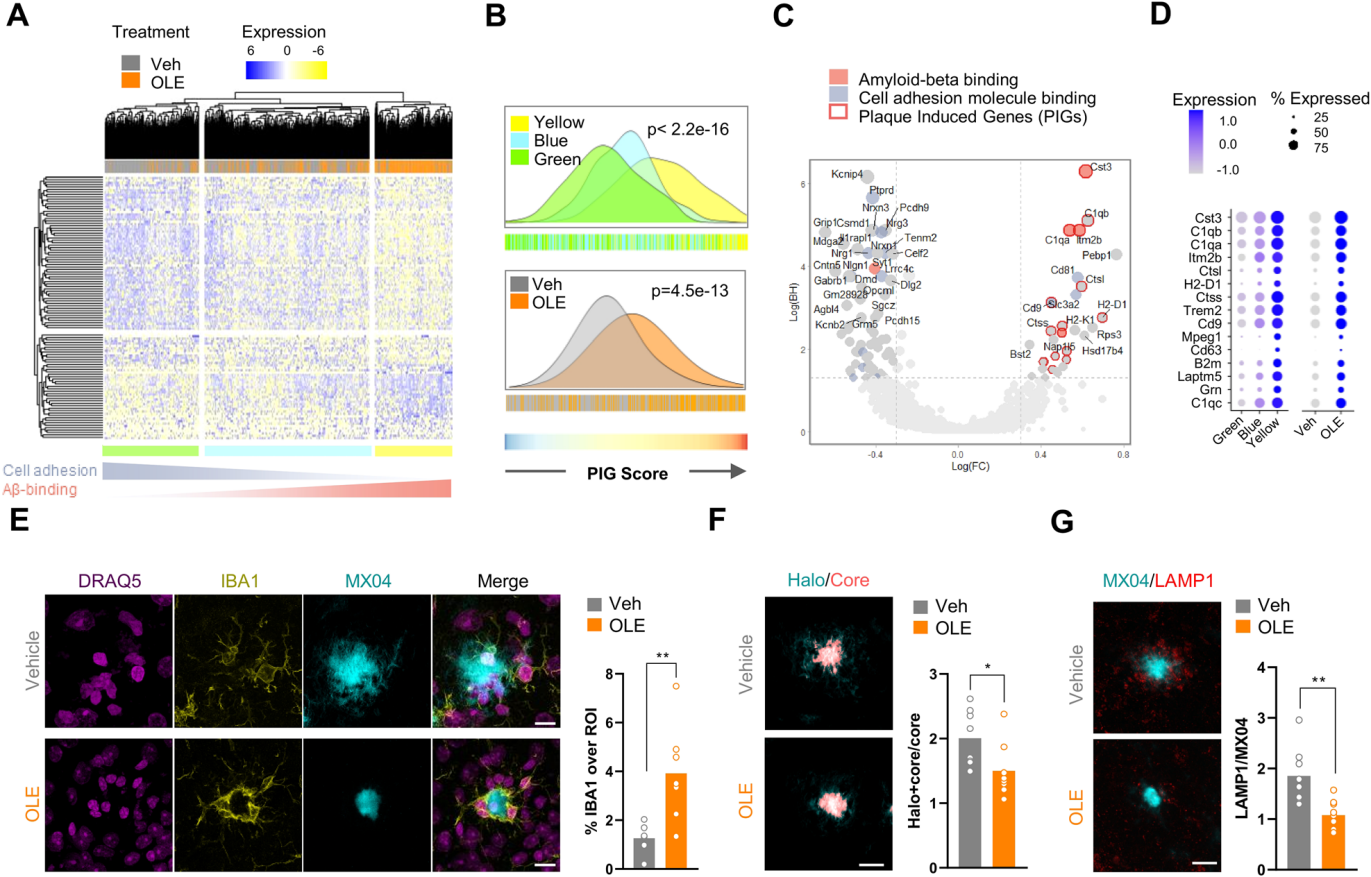
Microglia analysis in OLE treated APP/PS1 mice. **A** Heatmap clustering of microglia using OLE treatment DEGs. **B** Density plots of microglia according to the color-cluster, treatment and their relation with the PIG score; one-way ANOVA test. **C** Volcano plot showing microglia DEG in vehicle and OLE treated APP/PS1 mice. FC, Fold change. BH, Benjamini-Hochberg False Discovery Rate p-value correction. **D** Dot plot of the overlap between PIG and OLE upregulated and Yellow genes. **E** Left, representative IHC staining with methoxy-X04 (MX04, amyloid plaques) and Ionized calcium-binding adaptor molecule 1 (IBA1, microglia) of vehicle and OLE treated APP/PS1 mice. Scale bar, 50 µm. Right, quantification of IBA1 area within MX04 ROI. *n* = 6–7 per group, *p* = 0.0051; one-sided Student’s *t*-test. **F** Left, representative IHC image of amyloid plaque halo and dense core using low- and high-MX04 signal thresholds, respectively. Scale bar, 50 µm. Right, percentage of the halo area over the dense core. *n* = 8 per group, *p* = 0.0389; one-sided Student’s *t*-test. **G** Left, representative IHC image of MX04 and Lysosome-associated protein 1 (LAMP1) staining in amyloid plaques. Scale bar, 50 µm. Right, percentage of the LAMP1 area over to the dense core. *n* = 8–9 per group, *p* = 0.0132; one-sided Student’s t-test. Single values (animals) are represented by dots. Average values are represented by gray (vehicle) and orange (treatment) bars. *p < 0.05 and **p < 0.01.

To gain deeper insights into OLE-induced altered microglia functioning, we performed a deeper cluster analysis of microglia and identified three independent subclusters, corresponding to homeostatic, disease associated microglia (DAM), and infiltrating macrophage cells (Supplementary Fig. 6b). Note that the latter group was excluded for subsequent analysis due to the low number of cells (i.e., 22 cells). We found that DAM and homeostatic ratios were not significantly altered in OLE treated mice (i.e., 54% of DAM in vehicle vs 55% of DAM in OLE treated mice) (Supplementary Fig. 6b). Similar results were obtained when microglia were hierarchically clustered using DAM DEGs (Supplementary Fig. 6c) and when the levels of expression of DAM genes were compared between treated and vehicle microglia (i.e., DAM score, pval = 0.82), albeit with a trend in DAM expression when previously published DAM signatures [59] were used instead (Supplementary Fig. 6c,d). Conversely, when microglia were hierarchically clustered using treatment DEGs, three independent subclusters were observed, which we termed yellow, blue and green, with yellow being particularly enriched for treated cells (Fig. 3A). The yellow cluster was also enriched for cell-migration (“Glial cell migration” GO:0008347, pval 5.7 × 10^−4^) and plaque induced genes (PIGs) [60] (PIGs, pval 1.4 × 10^−95^) (Supplementary Table 8). Accordingly, when ranking cells using the levels of expression of PIGs, we observed that both yellow and treated cells showed higher PIG scores (Fig. 3B) and increased expression of a common set of plaque-induced genes, such as Complement C1q A Chain (*C1qa*) and Triggering Receptor Expressed on Myeloid Cells 2 (*Trem2*) (Fig. 3C, D). Together, these changes are suggestive of a stronger association of yellow and treated microglial cells with amyloid plaques.

To confirm this in silico association, we next performed a series of IHC assays. We stained amyloid plaques with Methoxy-X04 (MX04) and microglia with Ionized calcium-binding adaptor molecule 1 (IBA1). We measured the relative MX04 area occupied by IBA1, and observed an increased microglia association in OLE treated mice (Fig. 3E). Similar results were obtained when microglia were stained with CST3, the top microglia upregulated treatment DEG (Supplementary Fig. 7a). Then, we sought to determine whether the organization of amyloid plaques was also altered. Amyloid plaques are typically composed by a dense Aβ core surrounded by a halo of more soluble Aβ forms [61]. Microglia are known to be more effective at clearing the diffuse areas than the dense cores [62]. We observed that the relative size of the diffuse halo was particularly reduced in OLE treated mice (Fig. 3F, and Supplementary Fig. 7b, c). Next, since diffusible Aβ forms are also more reactive [4], we examined neuronal damage by staining with Lysosome-associated protein 1 (LAMP1), which is increased in dystrophic neurites associated with amyloid plaques [63], and observed a stronger LAMP1 area reduction in OLE treated mice (Fig. 3G, and Supplementary Fig. 7d,e). Together, these data suggest that OLE treatment effectively increases microglia association with amyloid plaques and reduces their size, number and toxicity.

### OLE treatment in vitro increases microglia Aβ chemotaxis and clearance, and neuronal viability against oxidative and unfolded protein stress

**Fig. 4:**
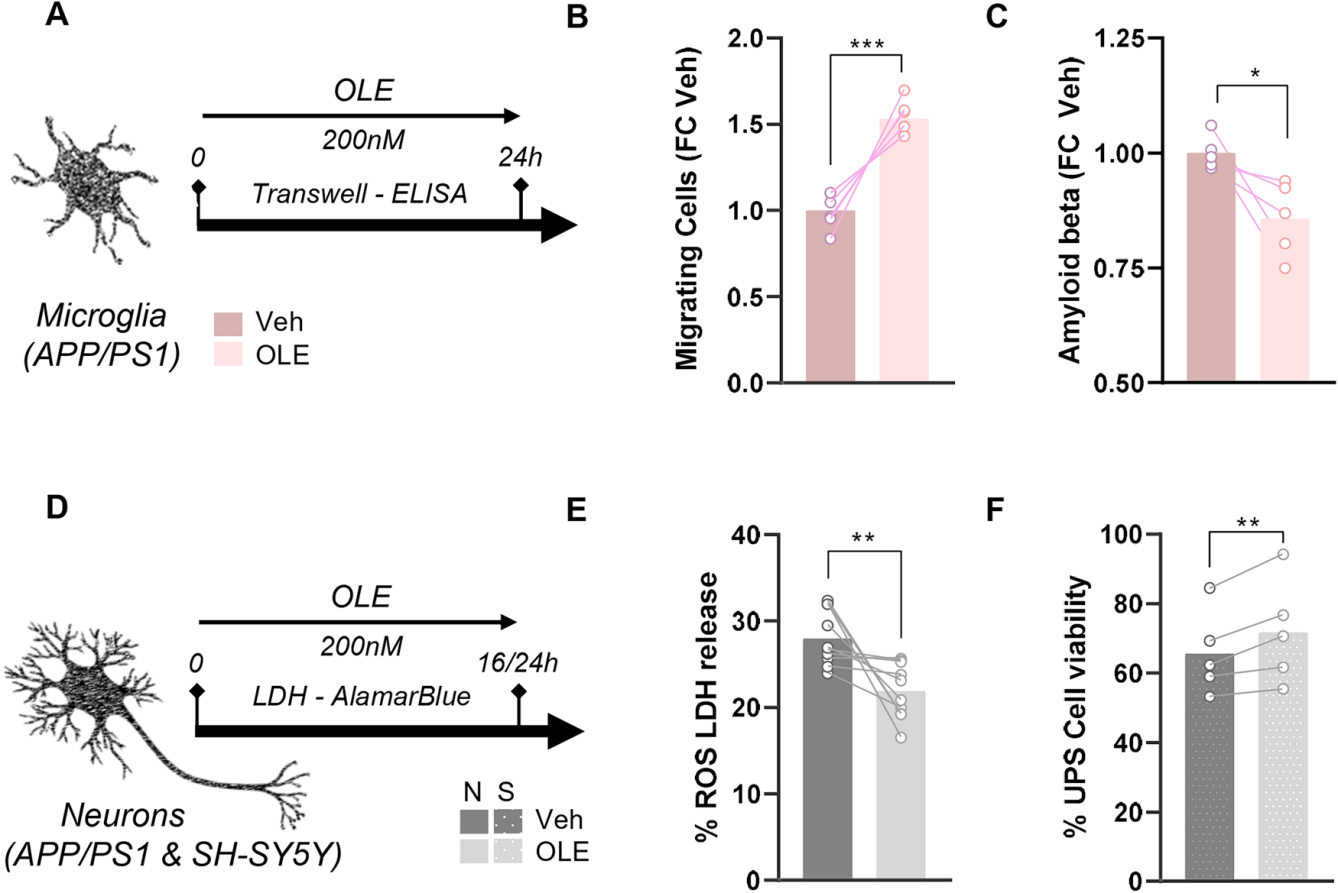
OLE treatment in microglia and neuronal cultures. **A** Schematics of microglia experiments. **B** Transwell assay of APP/PS1-derived primary microglia treated during 24 h with 200 nM OLE. *n* = 6 per group, *p* = 0.0007; one-sided Student’s *t*-test. **C** Amyloid-beta 40 (Aβ40) ELISA assays of APP/PS1-derived primary microglia cultures after 24 h of 200 nM OLE treatment. *n* = 5 per group, *p* = 0.0201; one-sided Student’s *t*-test. **D** Schematics of neuron experiments. N, primary hippocampal cultures derived from APP/PS1 mice. S, SH-SY5Y neuroblastoma cell lines. **E** LDH viability assay of APP/PS1-derived hippocampal neuronal cultures treated during 16 h with 150 µM H2O2 and 200 nM OLE. ROS, reactive oxygen species. *n* = 10 per group, *p* = 0.0048; one-sided Student’s *t*-test. **F** AlamarBlue cell viability assay of SH-SY5Y cells treated during 24 h with 10 µM thapsigargin and 200 nM OLE. UPS, unfolded-protein stress. *n* = 5 per group, *p* = 0.0084; one-sided Student’s *t*-test. Single values (experiments) are represented by dots. *p < 0.05, **p < 0.01, and ***p < 0.001.

To support these observations on a functional level, we then performed a series of in vitro assays in primary cultures of microglial and neuronal cells. First, we measured Aβ chemotaxis in microglia using a transwell migration assay [64]. Briefly, APP/PS1 microglia were plated on a chamber sealed with a permeable membrane and their migration to a second chamber containing Aβ quantified (Fig. 4A). In line with the in vivo results, OLE treatment increased Aβ chemotaxis in cultured microglia (Fig. 4B). Second, we investigated whether OLE treatment also affected Aβ levels by ELISA, and observed that OLE treatment increases Aβ clearance (Fig. 4C).

Next, to determine whether the protective effects of OLE were exclusively mediated by microglia cells, we evaluated ROS damage in neurons, which is induced by Aβ [65] and increased in the surroundings of amyloid plaques [66]. We measured cell viability using the lactate dehydrogenase (LDH) activity assay [67] (Fig. 4D), and observed that OLE treatment also increases cell viability when subjected to ROS stress (Fig. 4E). Similar results were also observed in neuroblastoma SH-SY5Y cells with the AlamarBlue viability assay [68] (Supplementary Fig. 8 a,b). Lastly, we investigated whether OLE treatment also protects neurons against unfolded-protein stress (UPS), in accordance with the enriched pathways and DEGs (Fig. 2G and Supplementary Table 7). We used thapsigargin, which induces UPS by reducing the calcium-dependent chaperone activity [69], and observed that OLE treatment also increases cell viability when exposed to UPS (Fig. 4F). Similar results were obtained when SH-SY5Y cells were treated with tunicamycin, an inhibitor of protein glycosylation [69], to induce UPS (Supplementary Fig. 8c). These findings stipulate that OLE induces cell-type specific changes, particularly in microglia, where it increases amyloid chemotaxis and clearance, and in neurons, where it enhances the protection against ROS and unfolded-protein stress.

### OLE, but no other NAAA, induces microglia-mediated Aβ clearance in vitro

To determine whether other PM20D1-related NAAA could exert similar protective effects, we tested a series of commercially available NAAA, focusing on compounds with a similar fatty acid chain length and degree of unsaturation, as these characteristics largely determine their functional activity [14]. We also included oleic acid and leucine as additional controls (Supplementary Fig. 9 a, b). We measured microglia Aβ clearance, as the most salient AD-related phenotype, and observed that OLE, but no other NAAA, oleic acid or leucine alone, induced a significant increase in Aβ clearance after 24 h of treatment in vitro (Supplementary Fig. 9 c,d). Interestingly, arachidonoyl Serine (C20:4-Ser), a potent mitochondrial NAAA uncoupler [14], did not modify microglia Aβ clearance, suggesting that the observed effects are specific of OLE and most likely driven by non-mitochondrial uncoupling related mechanisms.

### OLE RNA expression signatures are conserved in human postmortem samples

**Fig. 5:**
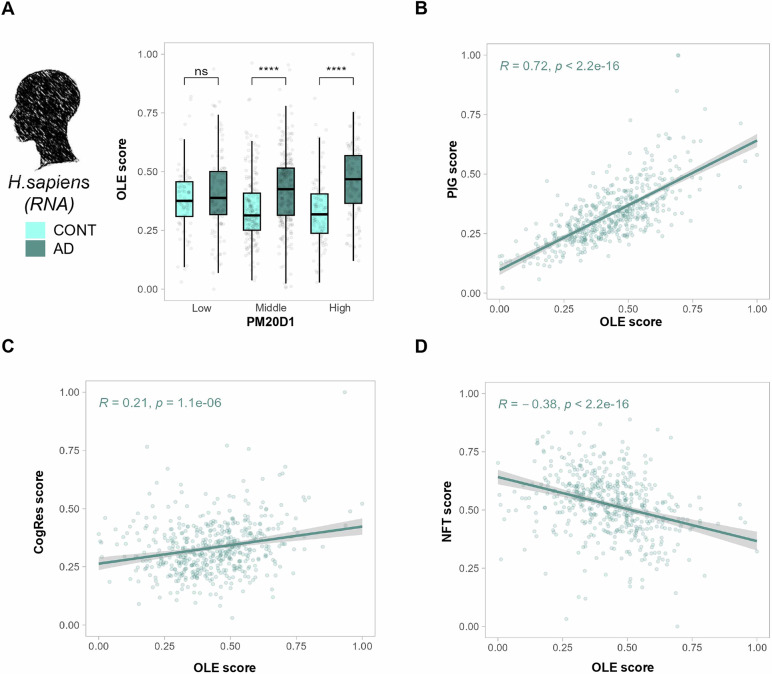
Human RNA expression signatures. **A** OLE score according to PM20D1 haplotype in AD and control postmortem brain samples. **B**–**D** Correlation between PIG, CogRes and NFT scores with OLE score in AD samples. a, *n* = 898, Wilcoxon test, ****p < 0.0001. **B**–**D**, *n* = 511, Spearman correlation. Data from GSE33000, GSE15222 and GSE48350.

Finally, to determine whether similar effects can be observed in human AD, we performed a series of transcriptomic analysis using publicly available data from postmortem AD brains (GSE33000, GSE15222 and GSE48350). First, since PM20D1 is upregulated in AD [8, 70], we tested if genes upregulated in mice were also increased in AD samples. We selected the DEGs represented in top identified pathways (Fig. 2E and Supplementary Table 9) and observed a significant increase of their expression in human AD brains, particularly in *PM20D1* high expression carriers (Fig. 5A and Supplementary Table 9). Similar results were obtained for top microglia DEGs, such as *CST3* (Supplementary Fig. 10a). Second, to investigate their association with amyloid plaques, we analyzed the expression of PIGs [60] as proxy of microglia interaction with amyloid plaques. OLE genes and PIG signatures were also highly correlated in human AD samples (Fig. 5B). Note that only non-overlapping genes were considered for the correlation. Similar results were obtained when alternative plaque-associated microglia markers, such as MER proto-oncogene tyrosine kinase (*MERTK*) [5] and C-Type Lectin Domain Containing 7 A (*CLEC7A*) [71] as well as when PM20D1 expression was considered instead (Supplementary Fig. 10b-f). Finally, to investigate whether OLE exert similar protective effects in human brains, we measured the expression of genes associated with cognitive reserve (CogRes) [72] and neurofibrillary tangle (NFT)-containing neurons [73] as proxies of cognition and neuronal damage, respectively, which are inversely correlated (Supplementary Fig. 10g). We found that CogRes genes were positively correlated with the expression of OLE genes, while NFT genes showed the opposite pattern (Fig. 5C, D and Supplementary Fig. 10h,i). Taken together, these results support the potential protective role of *PM20D1*/OLE in human AD.

## Discussion

Here, we show that the *PM20D1*-derived NAAA OLE improves AD pathologies in two independent models of AD, the GMC101 strain of *C. elegans* and the APP/PS1 mouse (Fig. 1), and provide mechanistic insights on its protective mode of action, namely by increasing microglia association with amyloid plaques and enhancing neuronal protection in mice (Figs. 2, 3 and Supplementary Fig. 6,7) and in vitro (Fig. 4 and Supplementary Fig. 8), with similar results in human AD patients (Fig. 5 and Supplementary Fig. 10), which, combined, support the protective role of PM20D1 in AD [8–13], and highlights the therapeutic potential of the OLE against AD.

PM20D1 was originally characterized as a bidirectional enzyme able to induce both, the synthesis and hydrolysis of NAAA. However, when PM20D1 is overexpressed in mice, it mostly increases NAAA levels [14] and PM20D1 and NAAA levels are also positively correlated in human PM20D1 expressing carriers [34]. Hence, PM20D1 activity in vivo seems to favor the condensation of NAAA, particularly of OLE [14, 34], which we used here. Note that PM20D1 can also facilitate the N-acylation of other species, such N-arachidonoyl dopamine, which protects against Parkinson’s disease [74]. Similar “reverse hydrolysis” has also been described for other NAAA-involved enzymes such as carnosine dipeptidase II (CNDP2), which favors the production of N-Lactoyl-Phenylalanine in vivo [75], and aminoacylase I (ACY1) and fatty acid amide hydrolase (FAAH) [40], which can also induce NAAA synthesis in vitro. Other enzymes such as Cytochrome P450 4F2 (CYP4F2) also participate in NAAA availability [76]. Actually, NAAA have previously been described as compounds with anti-inflammatory, neuroprotective and mitochondrial uncoupling activity [14, 37, 77]. They are increased by cold-exposure [14], sleep [78], and physical exercise [75], and enriched in nuts [79] and Korean fermented food Kimchi [80], all of which reduce inflammation [81–85], and improve AD pathologies and phenotypes [86–90]. NAAA might also exert protective effects against AD by mimicking the effects of caloric restriction [91] and ketogenic diet [92] through mitochondrial uncoupling [14], or by decreasing phenotypes associated with AD, such as obesity [14, 16–19] and diabetes [14, 15], through improvement of glucose and insulin metabolism [14].

Our results support the role of OLE in modulating inflammatory responses and, in particular, in modifying microglia (Figs. 2–4). We did not observe any significant enrichment in genes associated with mitochondria and/or metabolic activity, although the increase in the expression of *Atp2a2*, which is involved in autophagy in response to starvation [93], and the increase in *Hsd17b4*, which senses NAD+ levels [94], and regulates M1 to M2 macrophage transition [53], may indicate a compensatory effect to mitochondrial uncoupling in neurons and a metabolic shift in AD microglia [95], respectively (Fig. 2e). Genes such as *Hsp90ab1*, which protects neurons against unfolded protein stress [57], and *Slc3a2*, which increases microglia amyloid phagocytosis [54], were also up-regulated in OLE treated mice (Figs. 2e and 3c) which, together with ATP2A2 and HSD17B4, have also been shown to interact with NAAA [14]. Other pathology-relevant genes such as *Cst3* and *Itm2b*, whose mutations cause familiar forms of amyloidosis, and whose overexpression protects against AD [51, 52], and *Hspa5*, which protects against learning and memory deficits in TAU mouse models of AD [58], were also upregulated in APP/PS1 OLE treated mice. Whether these interactions are necessary and sufficient to protect against AD, or whether they can be exploited to improve current therapies for the disease deserves further attention now.

Although this study shows promising neuroprotective effects of an OLE treatment against AD, the following limitations and open questions remain. First, as the treatment was applied systemically, it can exert a series of primary and secondary actions. While our analysis focused on the most responsive cell types, it did not address the possibility of their interactions. Our results indicate that microglia association with amyloid plaques was correlated with the reduction of their size, but also with the decrease of the neuronal damage. The lack of overlap between DEGs from different cell types (Fig. 2B), the strong decrease in LAMP1 staining in vivo (Fig. 3G and Supplementary Fig. 7d, e) and the observed NAAA neuroprotection in vitro (Fig. 4D–F and Supplementary Fig. 8), indeed suggest the co-existence of other cell-type-specific effects. Whether OLE primarily affects microglia, with functional consequences on the rest, or whether it exerts distinct effects on different cells requires further investigations. Second, it remains unclear whether the OLE is the only neuroprotective one among the NAAA, which result of the conjugation of a myriad of fatty acids and amino acids. Here, we focused on OLE due to its good tolerance in vivo and the strong correlation between OLE and PM20D1 levels in humans [34] and mice [14]. While no other tested NAAA exhibited similar effects in vitro (Supplementary Fig. 9), additional NAAA may yield different outcomes, particularly in vivo. Finally, it remains to be determined whether OLE also protects against tau pathologies and neurodegeneration, as both the GMC101 strain of *C.elegans* and APP/PS1 mice lack these AD hallmarks. Notwithstanding, our data suggest that this may be the case, namely because of the higher cognition and lower NFT scores in human patients (Fig. 5) and because OLE treatment increases cell viability against ROS and unfolded-protein stress (Fig. 4), both of which participate in TAU pathology [96, 97]. Whether OLE treatment protects and/or modifies disease progression in human AD patients surely deserves further testing.

In sum, our results support the use of OLE as a therapeutic agent against AD and provide insights into its mechanism of protection, both of which may contribute to the development of more efficient therapeutic strategies to counteract the progression of the disease.

## Materials and methods

### Mice

APP/PS1 mice were acquired from The Jackson Laboratory (Bar Harbor, Maine, USA) (JAX, 005864) and breed in specific-pathogen free (SPF) conditions with a 12-h light–dark cycle, temperature 19–23 °C and humidity 40–70%. Cages were located in ventilated racks and equipped with non-aspen wood bedding and a paper roll. Mice had *ad libitum* access to food and water. Experimental mice were obtained from crosses between transgenic males with WT females. Littermates were distributed into cages so that all contained both genotypes, and cages randomly assigned to treatment or vehicle groups. Only males were used for consistency with our previous *PM20D1* research [8]. OLE (60 mg) and/or vehicle (1 ml DMSO) was administered in alkaline drinking water (250 ml) from 15 to 18 months of age. Mice were handled 5 min/day during five consecutive days the week before assaying cognition. For behavioral experiments, mice were placed in the experimental room one hour before testing. All experiments were conducted in the morning. The novel object recognition (NOR) test was conducted in an open field arena of 42 × 30 cm. On the first day, mice were habituated for 10 min to the open arena. On the second day, mice were placed for 10 min in the arena with two identical objects, and the time exploring each object was automatically recorded using Ethovision (Noldus, Wageningen, the Netherlands). On the third day, one of the two familiar objects was replaced by a novel object. The time exploring each of the objects was recorded and an object recognition index was calculated as the time spent exploring the novel object divided by the total time spent exploring the two objects (RI = time_novel_ / time_novel_ + time_familiar_ object). The Morris water maze (MWM) was conducted in a pool of 120 cm of diameter and virtually divided in four equivalent quadrants: north (N), south (S), east (E) and west (W), with a 1 cm submerged escape platform (12 cm in diameter) in the W quadrant. The pool was full of opaque water and surrounded by distal visual cues. Four trials per day during four consecutive days were performed. Starting position of the animals was alternated between trials and the inter-trial time maintained between days. Mice were allowed to swim for 120 s and were guided to the platform if they failed to find it during the trial time. On the fifth day, the escape platform was removed and animals were place in the opposite platform location. Cumulative and average distance to the platform were automatically recorded (Ethovision, Noldus) and used to calculate cognitive performance as recommended for aged mice [98, 99]. The experimenter was blinded to the mice’s genotype and treatment during the cognitive tests. The exclusion criteria were defined a priori to ensure consistency across behavioral assessments. For the NOR test, mice that failed to explore each object for at least 10 seconds were excluded. Experiment started with 12 WT vehicle, 11 WT treatment, 12 APP/PS1 treatment, and 16 APP/PS1 treatment. One WT vehicle and one APP/PS1 treatment mouse were excluded. For the MWM test, mice that failed to reach the platform during visual platform training or did not learn the task (e.g., exhibiting floating or thigmotaxis) were excluded. Experiment started with 12 WT vehicle, 11 WT treatment, 12 APP/PS1 treatment, and 16 APP/PS1 treatment. One WT treatment, two APP/PS1 vehicle, and two APP/PS1 treatment mice were excluded. Additionally, one WT and one APP/PS1 treatment mouse were found dead and not completed the MWM experiment. Body weight and overall health of mice was assessed weekly. Human endpoints included a reduction in body weight of more than 20% and obvious signs of pain-distress [100]. No experimental mouse met the human endpoint criteria. Upon completion of the behavioral tests, mice were sacrificed. One hemisphere was freshly dissected and snap frozen for molecular biology experiments and the other hemisphere dedicated to immunohistochemistry (IHC) assays. Total Aβ was measured using Amyloid beta 40 and 42 ELISA Kits (Invitrogen). All animal procedures were conducted according to the Switzerland’s and Spain’s licenses VD2875 and 2020/VSC/PEA/0151, respectively.

### C.elegans

*C. elegans* were cultured at 20 °C on nematode growth medium (NGM) agar plates seeded with *E. coli* strain OP50 unless stated otherwise. The strain used in this study was GMC101 (unc-54p::A-beta-1-42::unc-54 3′-UTR + mtl-2p::GFP) [41], which was provided by the Caenorhabditis Genetics Center (University of Minnesota). The strain CL2122 was outcrossed 3 times in the N2 background, and subsequently used in the control experiments reported herein. OLE was dissolved in DMSO and added just before pouring the plates. For phenotyping experiments, L4 worms were allowed to reach adulthood on the treatment plates. The worms were therefore exposed to the compound during adulthood until death, or up to 8 days. For protein analysis experiments, synchronized L1 worms were exposed to the compounds until harvest. Worms were harvested at day 1 to avoid any bias caused by worm’s death. To ensure a permanent exposure to the compound, plates were changed twice a week. Mobility assay was performed as described [101], starting from day 1 of adulthood, using the Movement Tracker software. For paralysis score, L4 worms were allowed to reach adulthood on the treatment plates. Worms were manually scored for paralysis after poking, as already described [41]. Worms that were unable to respond to any, repeated stimulation, were scored as dead.

### Primary cultures

Primary hippocampal neuron cultures derived from post-natal day 0 (P0) APP/PS1 mice were cultured in media consisting of Neurobasal (Invitrogen, Waltham, Massachusetts, USA), B27 supplement 4% (Invitrogen), L-glutamine (50 mM) (Invitrogen) and penicillin/streptomycin 1% (Invitrogen) (0.2 ml per well) on 96 wells plates (2.5 × 10^4^ cells per well) coated with Cultrex poly-L-lysine (Trevigen, Kampenhout, Belgium). On day in vitro 14 (DIV14) 200nM [14] OLE (Cayman, Ann Arbor, Michigan, USA) or vehicle (ethanol) was added, cells treated with 100 µM H_2_O_2_ during 16 h and cell viability measured by LDH release assay (Promega, Madison, Wisconsin, USA). Primary astrocyte-microglia cultures derived from P3 APP/PS1 mice were cultured in MEM media (Gibco, Grand Island, New York, USA) supplemented with 4% L-glutamine (50 mM) (Invitrogen), penicillin/streptomycin 1% (Invitrogen) and 10% FBS (Cultek, Madrid, Spain) on 75 cm^2^ flasks (2 × 10^6^ cells). After two weeks of culture, microglia were harvested by gentle shaking. For transwell experiments, microglia were seed on 24 well transwell plates (Corning) (6 × 10^4^ cells per well) in a total volume of 150 µl (upper chamber, insert 6,5 mm transwell 8,0 µm pet mem), with 800 µl of conditioned Aβ media obtained from primary cultures derived from APP/PS1 mice at DIV14 (lower chamber). OLE 200 nM or vehicle (ethanol) was added at the moment of seeding the cells. Next day, migrated cells were stained with DAPI, images acquired with the Motic AE31E microscope and quantified with Image J. For Aβ clearance experiments, microglia were seed on 96 well plates (2 × 10^4^ cells per well), coated with poly-D-lysine (Trevigen), and cultured with the media supplemented with GM-CSF 0.25% (Sigma, Burlington, Massachusetts, USA) during two days (0.2 ml). On day three, equal amounts of conditioned Aβ media (10 µl), plus 200 nM of OLE, C20:4-Ser (Cayman), C16:0-Asp (Cayman), C18:1-Gly (Cayman), Oleic acid (Cayman), Leucine (Cayman) or vehicle (ethanol, DMSO or water), was added on the cells. On the fourth day, media was collected and remaining Aβ measured using the Amyloid beta 40 ELISA Kit (Invitrogen Novex).

### Cell lines

SH-SY5Y human neuroblastoma cells were cultured in media consisting of 4.5 g/L glucose Dulbecco Modified Eagle Medium (DMEM) (Biowest, Nuaillé, France) supplemented with 15% FBS (Corning, Corning, New York, USA), 2 mM L-glutamine (Gibco) and 100 mg/ml penicillin/streptomycin (Gibco) (0.1 ml per well) on 96 wells plates (1 × 10^4^ cells per well). On the next day, 200 nM OLE or vehicle (DMSO) was added, cells treated with 10 µM Thapsigargin (Fisher Scientific, Waltham, Massachusetts, USA) or 150 µM Hydrogen peroxide (Fisher Scientific) during 24 h, and cell viability measured using AlamarBlue assay (Invitrogen). The SH-SY5Y cell line was obtained from the American Type Culture Collection (ATCC, CRL-2266) and was routinely screened for mycoplasma contamination on a monthly basis.

### Synthesis of N-Oleoyl-Leucine

To produce OLE, a solution of Leucine (1 eq.) in acetone and water (1:1) was mixed with K2CO3 (2 eq.) and oleoyl chloride (1.5 eq.) at 0 °C. Then, the mixture was stirred at room temperature overnight. The reaction was then acidified with 1 M HCl until pH4. The reaction mixture was extracted with ethyl acetate, and the organic layer was washed with brine (2 × 200 mL) and dried over anhydrous Na2SO4. The solvent was dried under reduced pressure, and the crude product was purified on silica gel. The product was eluted at 30% of ethyl acetate in hexane, and oleoyl leucine was obtained as a sticky white solid (yield ~60%). 1H Nuclear Magnetic Resonance (NMR) (400 MHz, CDCl3) δ 10.43 (s, broad, 1H), 6.19 (d, J = 8 Hz, 1H), 5.34-5.31 (m, 2H), 4.64-4.59 (m, 1H), 2.25 (t, J = 4 Hz, 2H), 2.01-1.97 (m, 4H), 1.72-1.57 (m, 5H), 1.28-1.25 (m, 20H), 0.95-0.85 (m, 9H). 13 C NMR (CDCl3, 100 MHz) δ 176.51, 174.23, 50.88, 41.25, 36.47, 31.91, 29.77, 29.72, 29.53, 29.25, 29.19, 29.17, 27.23, 27.19, 25.68, 24.91, 22.86, 22.69, 21.89, 14.12. Electrospray Ionization Mass Spectrometry (ESI-MS), calculated for C24H46NO3 [M + H] + : 396.34, found: 396.41. For the synthetic scheme, 1H and 13 NMR, and ESI-MS analysis see Supplementary Fig. 1.

### snRNA-Seq

Frozen brain tissue was homogenized in 6 mL Solution D (Sucrose 0.25 M, KCl 25 mM, MgCl2 5 mM, Tween20 0.1%, Tris-HCl 20 mM pH:7.5; plus 1U/µl RNase inhibitor [Promega]) on ice and centrifuged for 5 min at 500 g at 4°C. Pellets were resuspended in 4 ml Solution D, plus 2 ml Optiprep (Proteogenix, Schiltigheim, France), mixed gently, and centrifuged for 10 min at 1500 g at 4°C. Finally, pellets were resuspended in 1X PBS with 0.04% BSA (Merck. Darmstadt, Germany), samples filtered (20 µm) and diluted to 1000 nuclei per microliter. Entorhinal cortex samples from five mice were pooled. Libraries were constructed using Chromium SingleCell 3′Reagent Kit v3 chemistry (10X Genomics, Pleasanton, California, USA) and sequenced using the NextSEq. 500 (v2.5). Cellranger count (CellRanger v3.0.1) aligned FASTQ files to the mm10 pre-mRNA genome. Seurat (v4.0.4) was used to calculate quality-control metrics, remove doublets, normalize data (SCTransform) and UMAP clustering. Cell type prioritization was performed using Augur method. Differential expression analysis was performed for each cell type using the logistic regression framework in Seurat’s FindMarkers. P-values were corrected using the False Discovery Rate Benjamini-Hochberg method. Genes were considered differentially expressed when log fold-change was greater than 0.25 and adjusted p-value smaller than 0.05. Pathway analysis was performed using gProfiler [102]. PIG and DAM scores represent scaled values of the mean Log1p expression of corresponding genes.

### Immunohistochemistry

Immunohistochemical analysis were performed on OCT embedded brains sections. Briefly, brains were fixed with 4% paraformaldehyde (PFA) for 18 h, cryoprotected with saccharose 30% for 48 h, and OCT-embedded for sectioning at 5-30 µm using the Slee MNT Cryostat. Tissue sections were blocked with BSA 3% and 0,1% Triton X-100, and incubated with the following primary antibodies: CST3 (1:200, R&D systems, Minneapolis, Minnesota, USA, AF1238), IBA1 (1:1 000, FUJIFILM Wako, Osaka, Japan, 019-19741), and LAMP1 (1:100, BD biosciences, Franklin Lakes, New Jersey, USA, 553792), and the corresponding secondary antibodies. For CST3 and LAMP1 staining, primary antibodies were incubated at 4°C overnight. For IBA1 staining, primary antibody was incubated at room temperature in presence of 5% newborn calf serum. Amyloid plaques were stained with Methoxy-X04 (Abcam, Cambridge, UK). Images were acquired with the confocal microscopes Olympus FV1 000 IX81, Leica SPEll, and Zeiss LSM 880-Airyscan, and Thunder Imager Inverted Microscopy, and analyzed with ImageJ and Imaris Software.

### Western blot

For worms, a total of ≈1000 worms were collected and lysed by sonication with RIPA buffer containing protease and phosphatase inhibitors (Roche, Basel, Switzerland). For mice, entorhinal cortex samples were lysed by sonication with laemmli buffer (50 mM Tris, 2% SDS, 2% Glycerol, 0.02% Bromophenol blue, pH 6.9). The concentration of extracted protein was determined by using the Bio-Rad DC Protein Assay (Hercules, California, USA) and 5% Mercaptoethanol was added prior loading. Equal amounts of protein (15–30 µg/lane) were separated by SDS–PAGE (10–15%) and transferred onto nitrocellulose and polyvinylidene difluoride (PVDF) membranes (Amersham, GE Healthcare, Chicago, Illinois, USA), respectively. Ponceau S staining (Sigma-Aldrich) was used for monitoring protein loading and transference. Nonspecific binding was blocked by incubation in 5% nonfat milk (worms) or 5% bovine serum albumin (mice) in phosphate-buffer saline containing 0.1% Tween (PBS) for 1 h at room temperature. Membranes were incubated overnight at 4 °C with the primary antibody rabbit anti-APP (6E10, Biolegend; Y188, Abcam) in PBS with 3% nonfat milk or 5% BSA in each case, and 1 h in the corresponding horseradish-peroxidase-conjugated secondary antibody. Immunocomplexes were revealed by an enhanced chemiluminescence reagent (ECL Advance, Amersham, GE Healthcare). Densitometric quantification was carried out by using ImageJ software. Each immunoblot experiment containing was repeated six times.

### RNA expression

RNA expression data from control and Alzheimer’s disease postmortem brain samples were obtained from the NCBI Gene Expression Omnibus (GEO) database (GSE33000, GSE15222 and GSE48350) and analyzed using R software (http://www.R-project.org). Z-score normalization was applied prior to merging. Samples were stratified in three segments according to the corresponding high, mid, and low expression PM20D1 haplotype – using the centroid-based clustering (K-means) method – as previously described [8]. N-oleoyl-L-Leucine (OLE), Plaque-induced gene (PIG), cognitive reserve (CogRes) and neurofibrillary tangle (NFT) scores were calculated as the average of the scaled expression values (range 0 and 1) of the corresponding genes. OLE genes were obtained from Fig. 2e (i.e., upregulated genes in biologically relevant pathways). PIG genes were obtained from Table S3 (Purple module) Chen et al. [60]. CogRes genes were obtained from TableS2 Yegla and Foster [72]. NFT genes were obtained from TableS2 (upregulated genes in at least four of excitatory clusters 1, 2, 3, 7 and 10) Otero-Garcia et al. [73]. The datasets obtained from the GEO database, were fully anonymized, and therefore did not require ethics approval.

### Study design and Statistical analysis

This study aims to provide evidence and mechanistic insight of PM20D1-OLE protection against AD, using a combination of GMC101 *C.elegans* and APP/PS1 mouse animal models, and behavioral, molecular and single-cell RNA sequencing tests. This approach concluded that PM20D1-OLE protects against AD through a mechanism that implies a higher microglia association with amyloid plaques. These results were then validated using IHC, in vitro experiments, and publicly available human postmortem RNA datasets. For all the experiments, sample sizes were determined on the basis of previous experience [8, 44]. For each experiment, the number of experimental unique replicates is specified in the figure legend. Behavioral and molecular analysis were performed by investigators blinded to genotype/treatment. Gene expression and in vitro analysis were performed unblinded. Statistical analysis was done using R software and Prism 8.0 (GraphPad, Boston, Massachusetts, USA) as described in the figure legends. Normality and homogeneity of variance were tested using Shapiro-Wilk and Levene’s tests. All experimental data points were included in the analysis.

## Supplementary information

Revised supplementary figures 1-10

Supplementary tables 1–9

Original Data

Full length uncropped original western-blot membrane used in Supplementary figure 3

Full length uncropped original western-blot membrane used in Supplementary figure 5

## Data Availability

The raw data generated in this study is available in the Gene Expression Omnibus repository (GSE214352) and the analysis code upon request.
