# Supplementary material for: The PM20D1-OLE pathway induces microglia rewiring to ameliorate Alzheimer disease
Source: Cell Death Dis. 2026 Apr 27;17(1):561. doi: 10.1038/s41419-026-08791-1 (PMC13254073; doi:10.1038/s41419-026-08791-1)
Supplement: Supplementary file 1 — Revised supplementary figures 1-10 [file 41419_2026_8791_MOESM1_ESM.pdf]

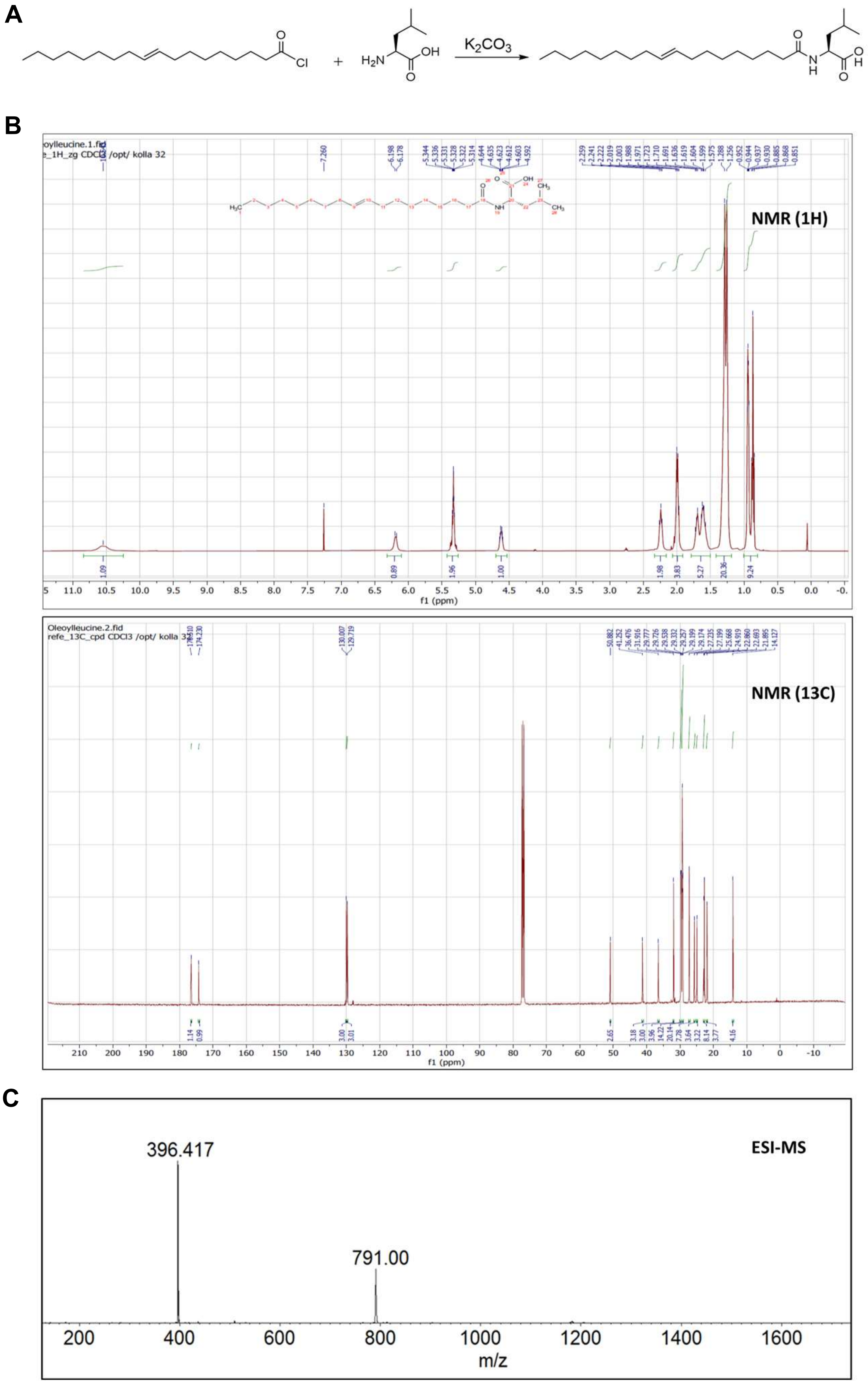

Supplementary Fig. 1 | Synthesis of N-Oleoyl-Leucine. (**A**) Synthetic scheme. (**B**) Nuclear Magnetic Resonance (NMR) analysis of N-Oleoyl-Leucine, 1H NMR (top) and 13C NMR (bottom). (**C**) Electrospray Ionization Mass Spectrometry (ESI-MS) analysis of N-Oleoyl-Leucine.

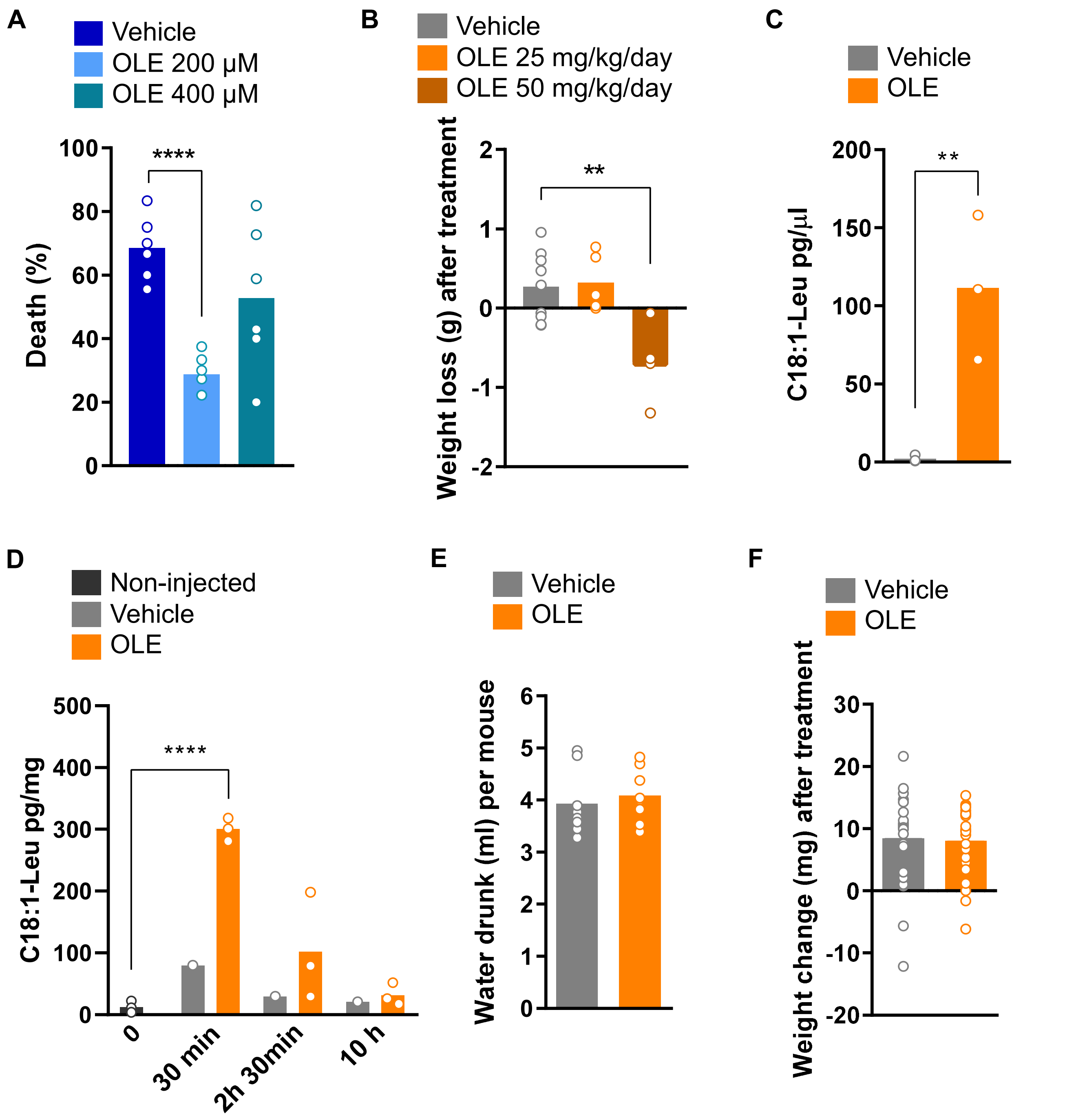

Supplementary Fig. 2 | OLE dose, absorption, blood brain barrier (BBB) and consumption tests. (**A**) GMC101 *C.elegans* survival at day 8 in presence of vehicle (DMSO) and OLE at 200 and 400μM. For death score, 45 L4 worms were allowed to reach adulthood on the treatment plates. Experiment was repeated six times. $p<0.0001$. (**B**) APP/PS1 mouse weight loss after two weeks of treatment with vehicle, 25 or 50 mg/kg/day OLE. n=4-10 per group, $p=0.0016$. (**C**) OLE pg per μl of plasma after 1 h of per oral (p.o.) administration of OLE 25mg/kg and/or vehicle (DMSO) measured by UHPL Exion LC Series (SCIEX) associated with a TripleTOF 6600+. n= 3 samples per group. $P=0.0075$. (**D**) OLE pg per mg of tissue (entorhinal cortex) after 30 min, 2 h and 30 min and 10h of intraperitoneal (i.p.) injection of OLE 25mg/kg and/or vehicle (DMSO). n= 3 samples per group. $p<0.0001$. (**E**) No differences in water consumption were observed in APP/PS1 mice after three months of 25 mg/kg/day OLE treatment. n=8 per group, $p=0.5976$; two-sided Student's t-test. (**F**) No differences in body weight were observed in APP/PS1 mice after three months of 25 mg/kg/day OLE treatment. n=27 per group, $p=0.8182$; two-sided Student's t-test. *$p<0.05$, **$p<0.01$, ***$p<0.001$, and ****$p<0.0001$ one-sided Student's t-test.

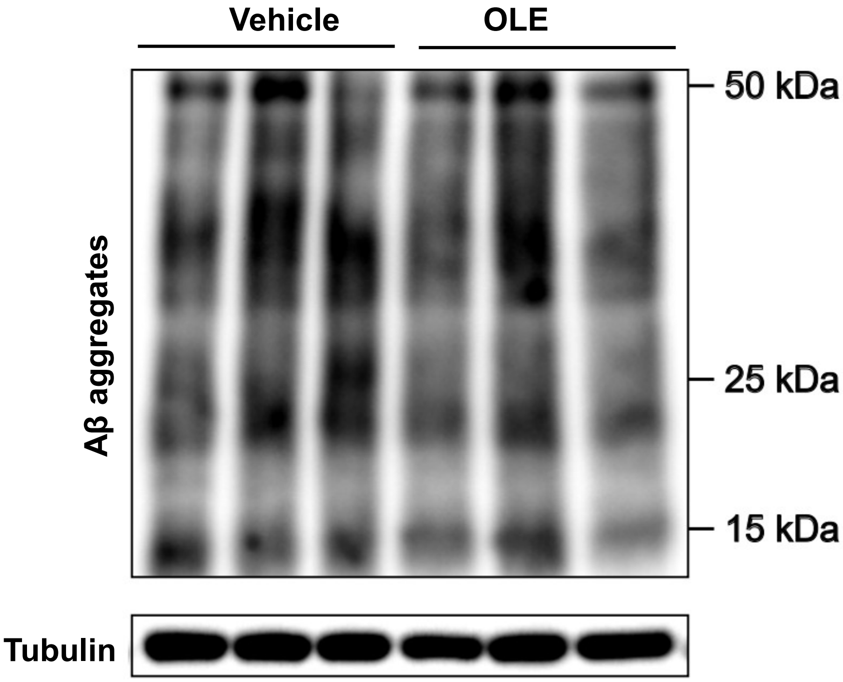

Supplementary Fig. 3 | Representative western-blot (WB) of amyloid beta (Aβ) aggregation in OLE and vehicle treated GMC101 *C. elegans.* Each WB line represents one vehicle or treated plate containing ~1000 worms.

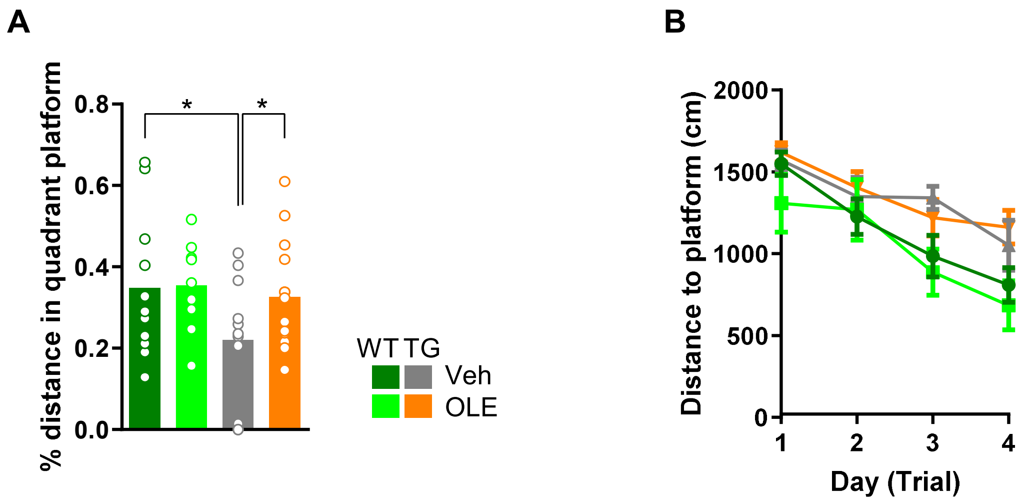

Supplementary Fig. 4 | OLE treatment characterization in APP/PS1 transgenic (TG) mice. (**A**) Similar differences between wild-type (WT) and vehicle and OLE treated APP/PS1 (TG) mice were observed when distance in the right quadrant platform was considered in the memory phase of the Morris Water Maze (MWM). n=9-14 per group, p=0.0445 for TG vehicle vs WT vehicle and p=0.0413 for TG treatment vs TG vehicle; one-sided Student's t-test. (**B**) No differences were observed in the learning phase of the MWM between OLE and vehicle treated APP/PS1 mice. n=11-15 per group, p= 0.8034; two-way ANOVA test. Single values represent different animals. *p<0.05.

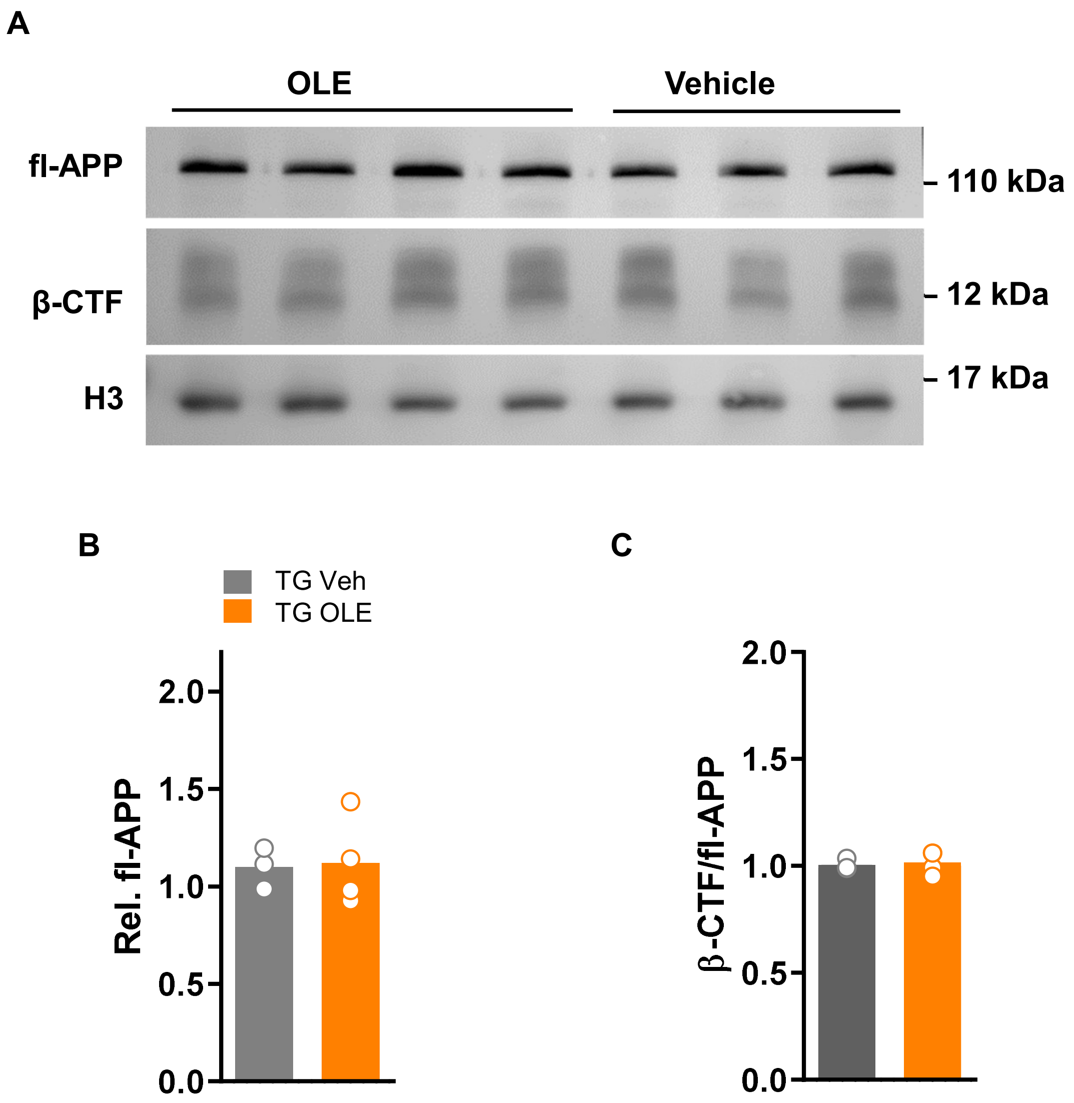

Supplementary Fig. 5 | APP levels and processing in OLE treated APP/PS1 mice. (**A**) Western-blot (WB) analysis of APP expression and cleavage using APP Y188 antibody. Equal amounts of protein (20 µg/lane) were separated by SDS–PAGE (15%) and transferred onto polyvinylidene difluoride (PVDF) membranes of 0.2µm (Amersham, GE Healthcare) using BioRad wet-transfer system for 18 h at 12V. Ponceau S staining (Sigma-Aldrich) was used for monitoring protein loading and transference. Nonspecific binding was blocked by incubation in 5% bovine albumin serum (BSA) in phosphate-buffer saline containing 0.1% Tween (PBS) for 1 h at room temperature. Membranes were incubated overnight at 4 °C with the primary antibody rabbit anti-APP (Y188, abcam) in PBS with 3% BSA and 1 h in the corresponding horseradish-peroxidase-conjugated secondary antibody. Immunocomplexes were revealed by an enhanced chemiluminescence reagent (ECL Advance, Amersham Biosciences). Densitometric quantification was carried out by using ImageJ software. Each immunoblot experiment containing was repeated three times. (**B**) Densitometric analysis of full-length (fl-APP) in (a). (**C**) Ratio between beta C-terminal APP fragment (β-CTF) and fl-APP in (A). n= 4 samples per group. p=0.8904 and p=0.7804; one-sided Student's t-test for (B) and (C), respectively. Histone 3 (H3) is used as loading control.

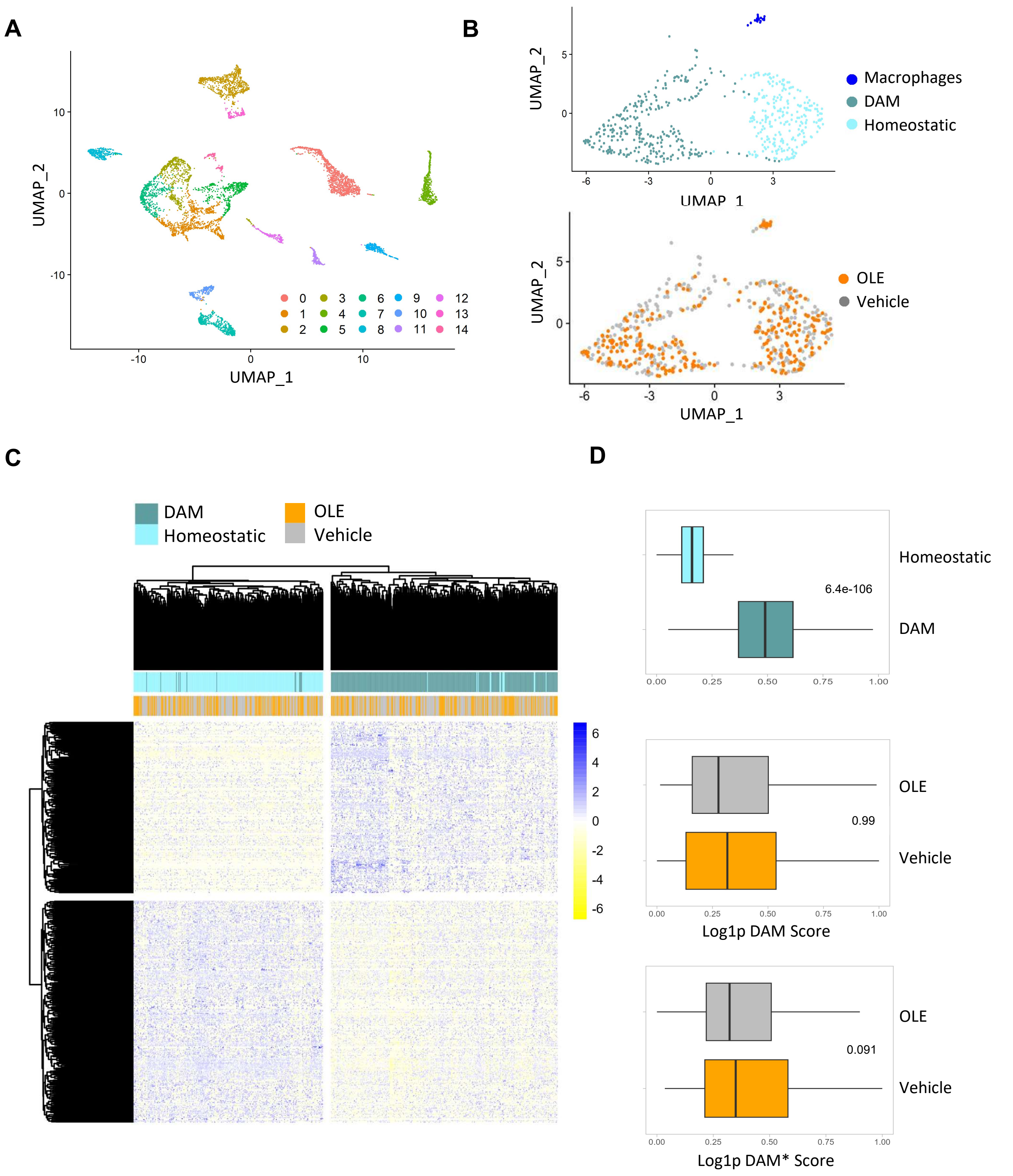

Supplementary Fig. 6 | Single-nuclei RNA sequencing (snRNAseq) characterization of entorhinal cortex samples obtained from vehicle and OLE treated APP/PS1 mice. (**A**) UMAP clustering colored by cell cluster. (**B**) UMAP clustering of microglia colored according to the identified subclusters (top) and treatment (bottom); Homeostatic microglia, disease associated microglia (DAM) and macrophages. (**C**) Heatmap clustering of microglia using DAM vs Homeostatic DEGs. (**D**) DAM score – as a proxy of DAM phenotype – comparison between Homeostatic and DAM cells (top) and between OLE and vehicle treated microglia (middle) using our DAM signature. Bottom, OLE and vehicle treated microglia DAM score comparison using published Keren-Shaul (*) et al signature[57]; two-sided Student's t-test.

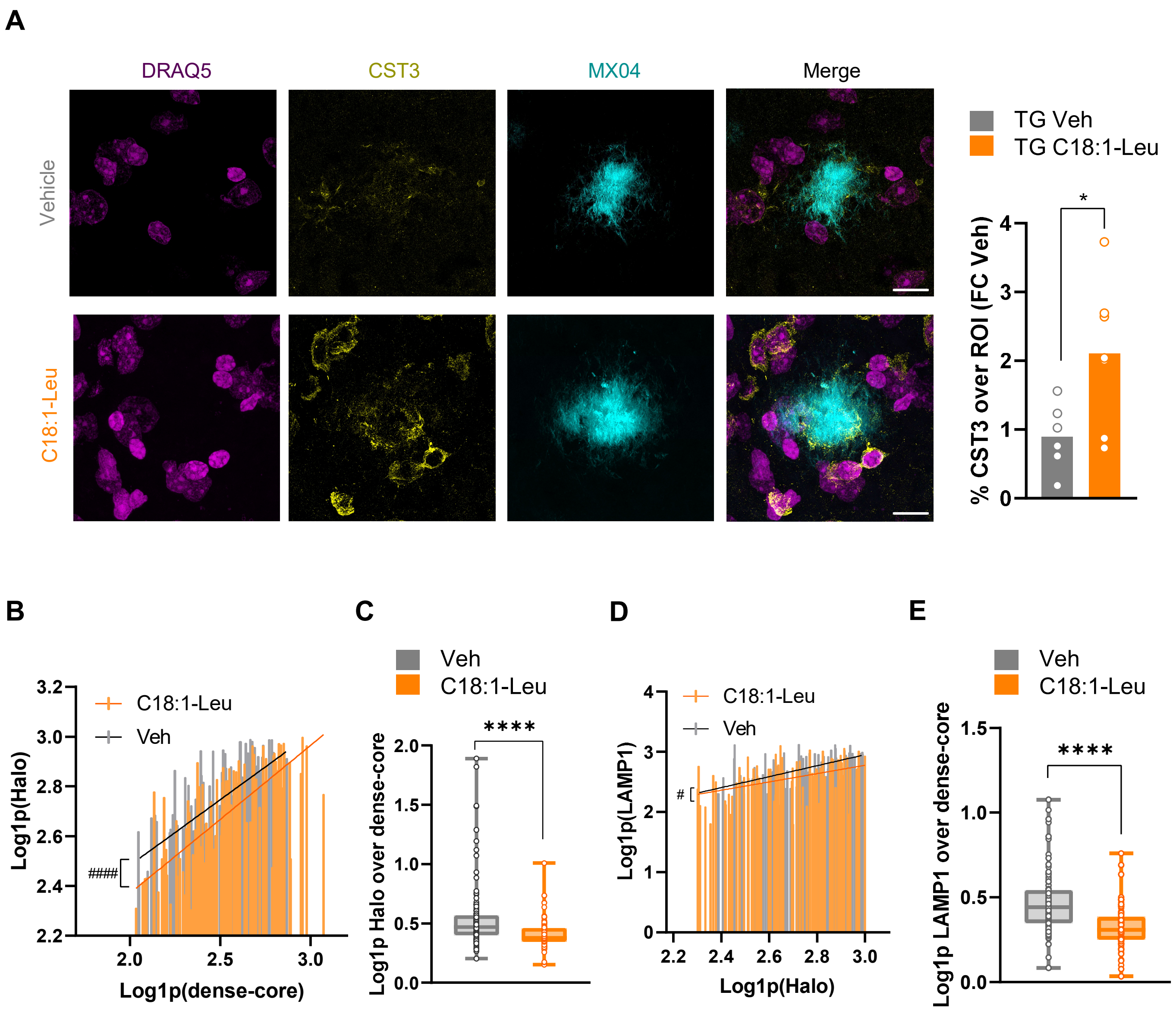

Supplementary Fig. 7 | Microglia IHC analysis in OLE treated APP/PS1 mice. (**A**) Left, representative IHC staining with MX04 and Cystatin 3 (CST3) of vehicle and OLE treated APP/PS1 mice. Scale bar, 50 µm. Right, quantification of CST3 area within MX04 ROI. n=6-7 per group, p=0.0132. (**B**) Comparison of amyloid dense-core and halo correlation in small- and medium-size (200-1000µm^2) amyloid plaques between OLE and vehicle treated APP/PS1 mice. n~150 per group, p<0.0001. (**C**) Comparison of the percentage of the halo area over the dense-core area in small- and medium-size amyloid plaques between OLE and vehicle treated APP/PS1 mice. n~120 per group, p<0.0001. (**D**) Comparison of amyloid plaque halo and LAMP1 staining correlation in small- and medium-size amyloid plaques between OLE and vehicle treated APP/PS1 mice. n~90 per group, p=0.0113. (**E**) Comparison of the percentage of the LAMP1 area over the dense-core area in small- and medium-size amyloid plaques between OLE and vehicle treated APP/PS1 mice. n~140 per group, p<0.0001. Single values in A represent different animals. Single values in B-E represent different plaques, in columns (B,D) and dots (C,E). #p<0.01, ####p<0.0001 linear regression slope intercept. *p<0.05, ****p<0.0001 one-sided Student's t-test.

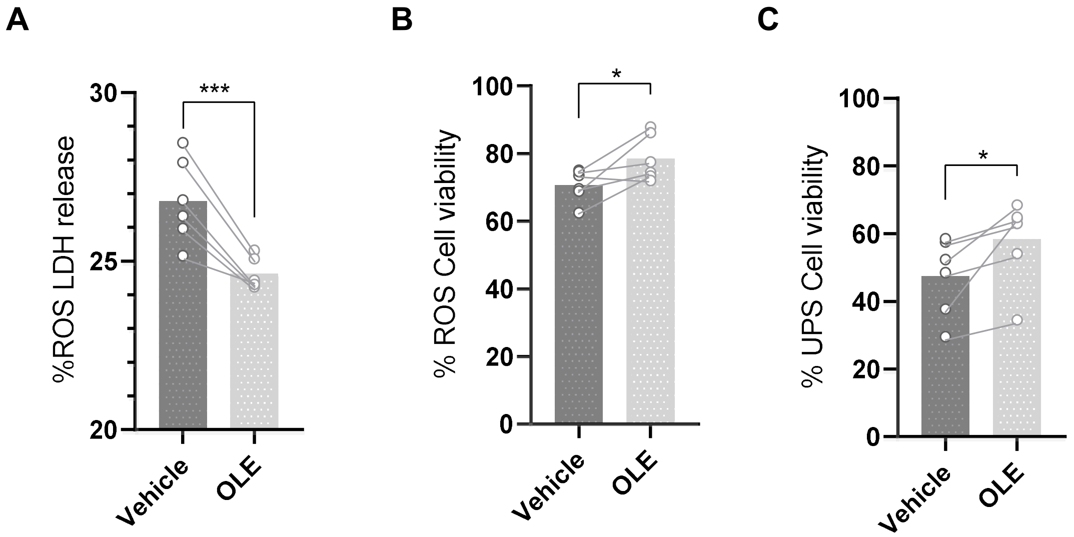

Supplementary Fig. 8 | OLE-mediated neuroprotection. (**A**) Lactate dehydrogenase (LDH) cell viability assays in SH-SY5Y neuroblastoma cells treated with 150µM H2O2 during 24h with OLE or vehicle. ROS, reactive oxygen species. n=6 per group, p=0.0009. (**B**) AlamarBlue cell viability assays in SH-SY5Y cells treated with 150µM H2O2 during 24h with OLE or vehicle. n=6 per group, p=0.0198. (**C**) AlamarBlue cell viability assays in SH-SY5Y cells treated with 30µg/ml Tunicamycin (MP Biomedicals) during 24h with OLE or vehicle. UPS, unfolded-protein stress. n=6 per group, p=0.0149. Single values (experiments) are represented by dots. *p<0.05, **p<0.01 and ***p<0.001, one-sided Student's t-test.

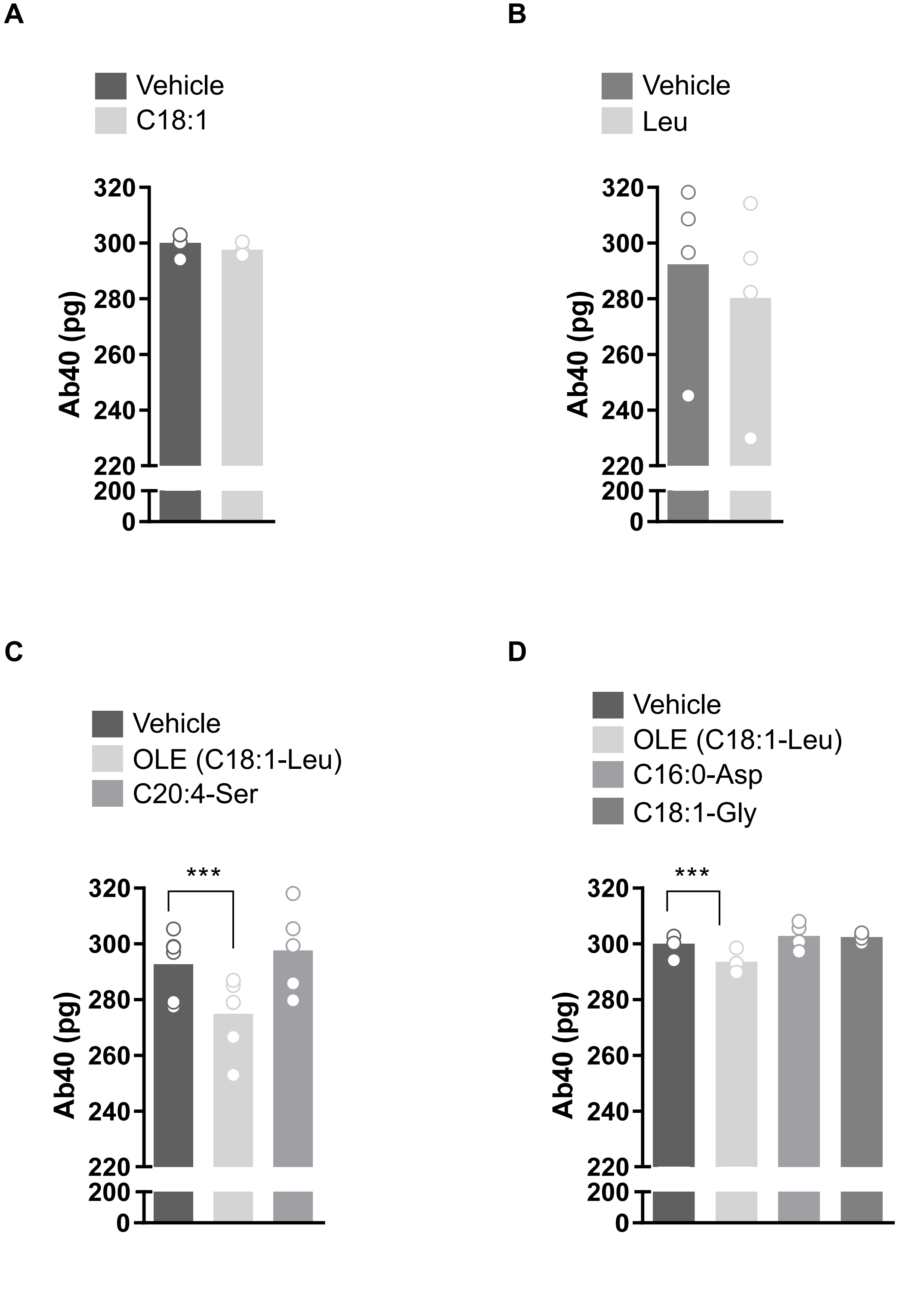

Supplementary Fig. 9 | PM20D1-derived NAAA treatments in primary cultures of microglia and hippocampal neurons derived from APP/PS1 mice. (**A**) Amyloid-beta 40 (Aβ40) ELISA assays of APP/PS1-derived primary microglia cultures after 24h of 200nM Oleic acid treatment diluted in DMSO. (**B**) Aβ40 ELISA assays of APP/PS1-derived primary microglia cultures after 24h of 200nM Leucine treatment diluted in water. (**C**) Aβ40 ELISA assays of APP/PS1-derived primary microglia cultures after 24h of 200nM C18:1-Leu (OLE) or C20:4-Ser treatment diluted in ethanol. (**D**) Aβ40 ELISA assays of APP/PS1-derived primary microglia cultures after 24h of 200nM C18:1-Leu, C16:0-Asp or C18:1-Gly treatment diluted in DMSO. n=4-8 per group, one-sided Student's t-test. Single values (experiments) are represented by dots. ***$p<0.001$.

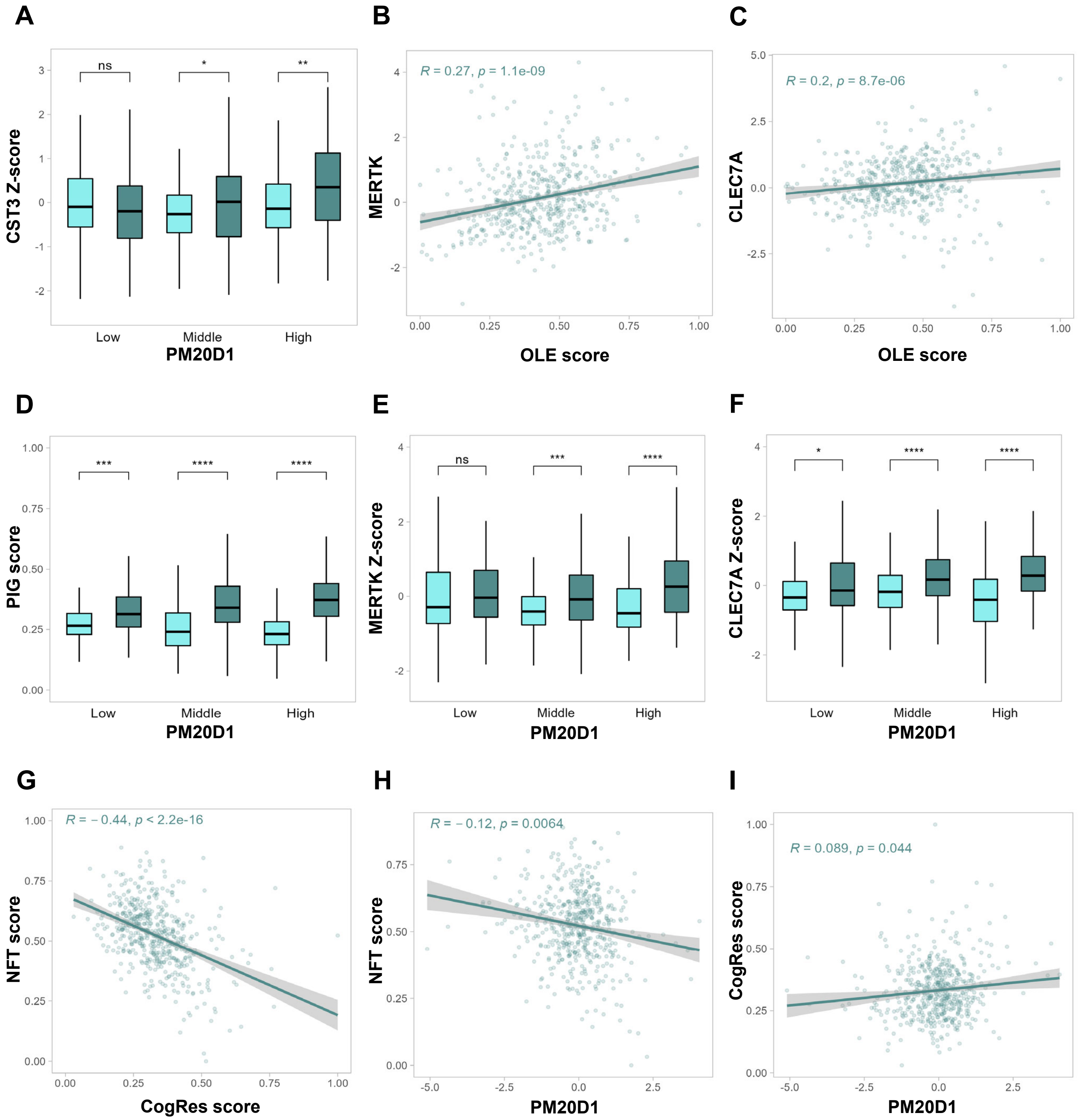

Supplementary Fig. 10 | RNA expression analysis in human samples. (**A**) *CST3* RNA expression in Alzheimer's disease (AD) and control postmortem brain samples according to *PM20D1* haplotype. (**B**),(**C**), OLE score correlation with *MERTK* and *CLEC7A* RNA expression levels in AD samples, respectively. (**D**),(**E**),(**F**), PIG score, *MERTK* and *CLEC7A* RNA expression in AD and control samples according to *PM20D1* haplotype, respectively. (**G**), CogRes and NFT scores correlation in AD samples. (**H**),(**I**), *PM20D1* RNA expression correlation with NFT and CogRes scores, respectively. A-D, n=898, Wilcoxon test, *p<0.05, **p<0.01, ***p<0.001, and ****p<0.0001. E-I, n=511, Spearman correlation. Data from GSE33000, GSE15222 and GSE48350.
