# Supplementary figures and images for: The PM20D1-OLE pathway induces microglia rewiring to ameliorate Alzheimer disease
Source: Cell Death Dis. 2026 Apr 27;17(1):561. doi: 10.1038/s41419-026-08791-1 (PMC13254073; doi:10.1038/s41419-026-08791-1)

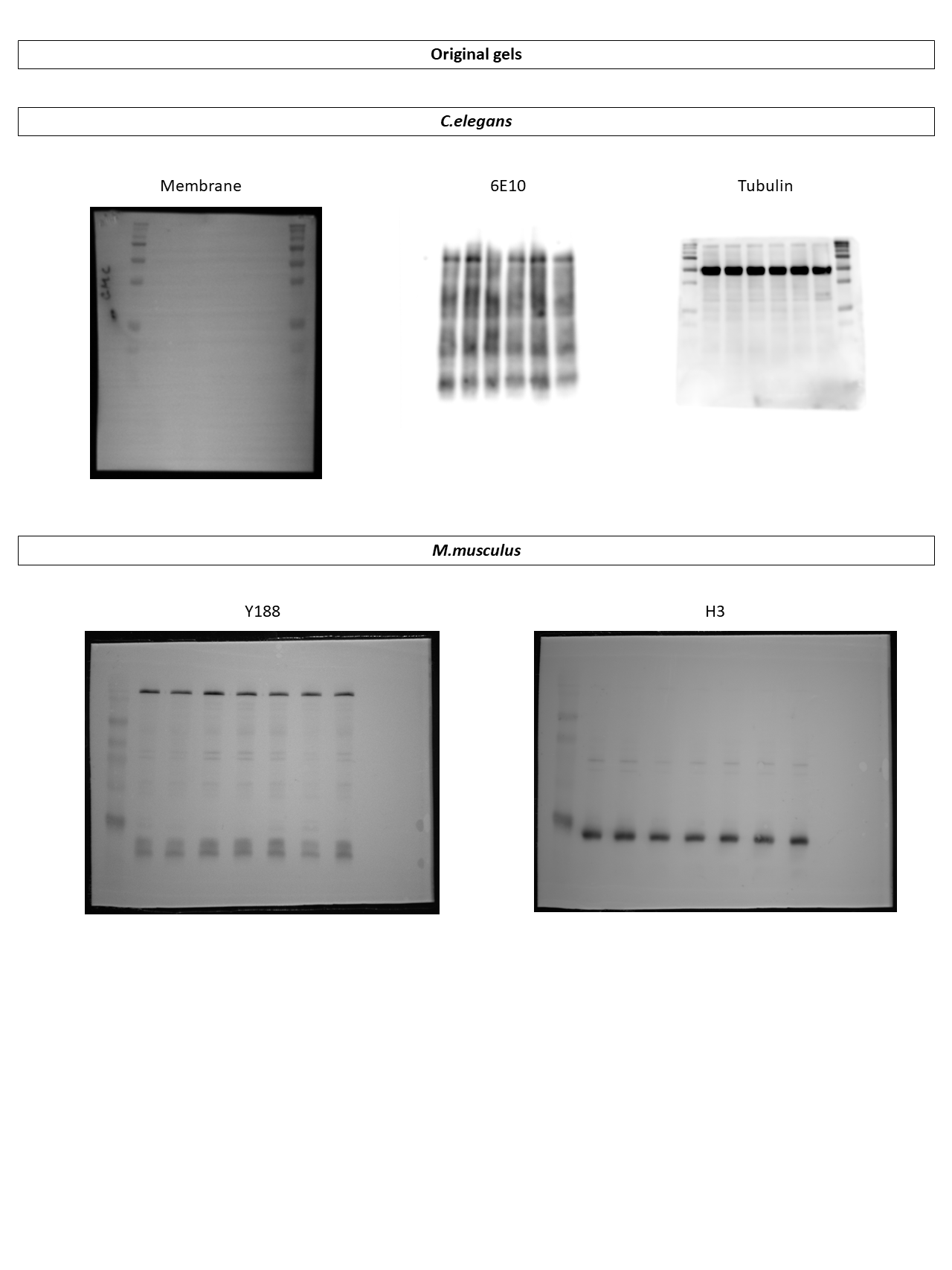

Supplement: Supplementary file 3 — Original Data [file 41419_2026_8791_MOESM3_ESM.tif]

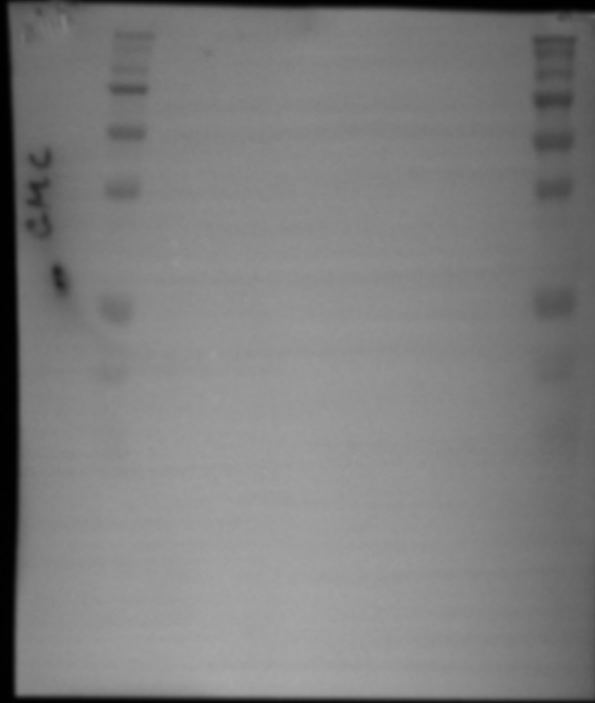

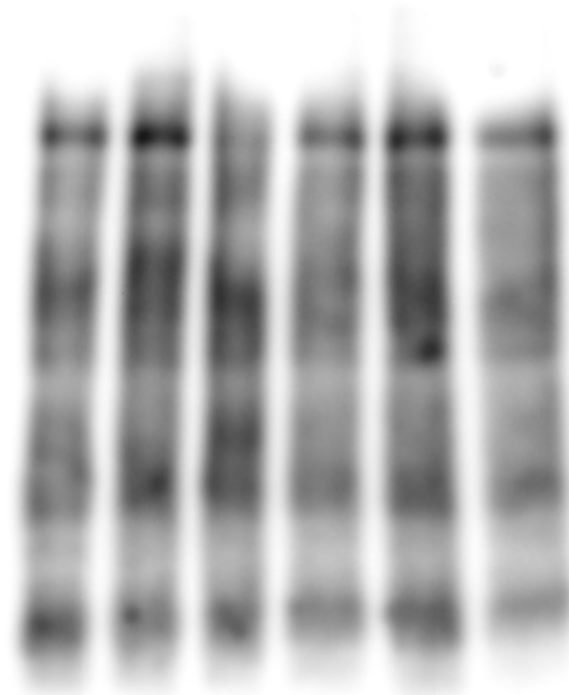

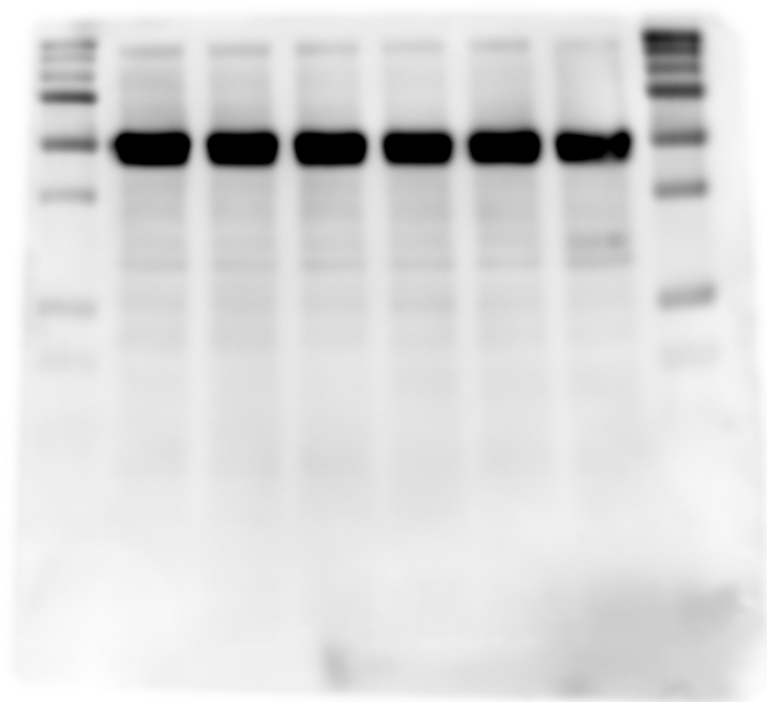

Supplement: Supplementary file 4 — Full length uncropped original western-blot membrane used in Supplementary figure 3 [file 41419_2026_8791_MOESM4_ESM.pdf]

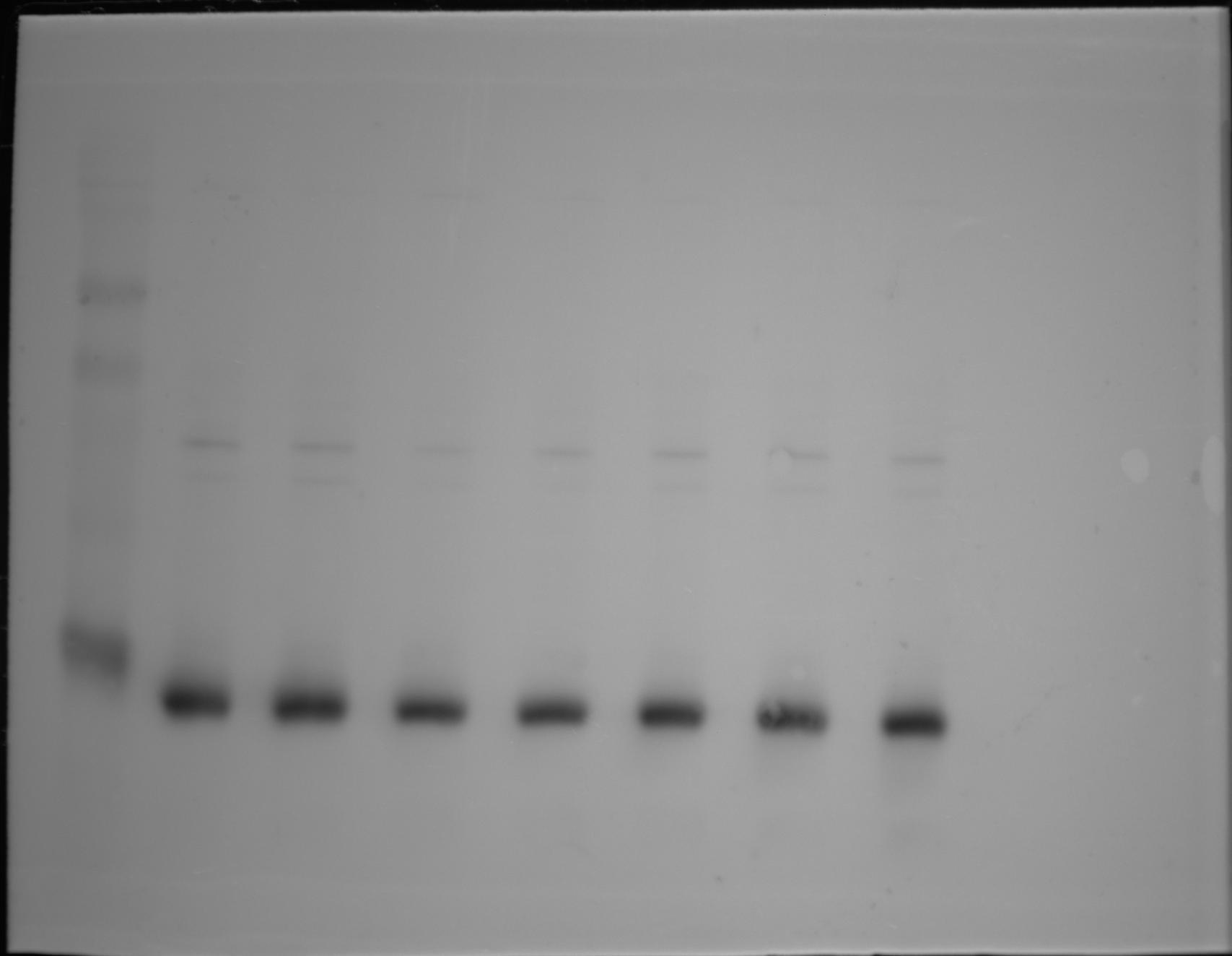

Supplement: Supplementary file 5 — Full length uncropped original western-blot membrane used in Supplementary figure 5 [file 41419_2026_8791_MOESM5_ESM.pdf]
